# Safety and efficacy of novel malaria vaccine regimens of RTS,S/AS01B alone, or with concomitant ChAd63-MVA-vectored vaccines expressing ME-TRAP

**DOI:** 10.1038/s41541-018-0084-2

**Published:** 2018-10-09

**Authors:** Tommy Rampling, Katie J. Ewer, Georgina Bowyer, Nick J. Edwards, Danny Wright, Saranya Sridhar, Ruth Payne, Jonathan Powlson, Carly Bliss, Navin Venkatraman, Ian D. Poulton, Hans de Graaf, Diane Gbesemete, Amy Grobbelaar, Huw Davies, Rachel Roberts, Brian Angus, Karen Ivinson, Rich Weltzin, Bebi-Yassin Rajkumar, Ulrike Wille-Reece, Cynthia Lee, Chris Ockenhouse, Robert E. Sinden, Stephen C. Gerry, Alison M. Lawrie, Johan Vekemans, Danielle Morelle, Marc Lievens, Ripley W. Ballou, David J. M. Lewis, Graham S. Cooke, Saul N. Faust, Sarah Gilbert, Adrian V. S Hill

**Affiliations:** 1 The Jenner Institute, University of Oxford, Oxford, OX3 7DQ UK; 2 NIHR Wellcome Trust Clinical Research Facility, University of Southampton and University Hospital Southampton NHS Foundation Trust, Southampton, UK; 3 Department of Medicine, Division of Infectious Diseases, University of California, Irvine, CA 92697 USA; 4 PATH Malaria Vaccine Initiative, Washington, DC USA; 5 Department of Life Sciences, Imperial College London, London, UK; 6 Centre for Statistics in Medicine, University of Oxford, Oxford, UK; 7 GSK Vaccines, Rixensart, Belgium; 8 Clinical Research Centre, University of Surrey, Guildford, GU2 7XP UK; 9 Infectious Diseases Section, Faculty of Medicine, Department of Medicine, Imperial College London, London, UK

## Abstract

We assessed a combination multi-stage malaria vaccine schedule in which RTS,S/AS01B was given concomitantly with viral vectors expressing multiple-epitope thrombospondin-related adhesion protein (ME-TRAP) in a 0-month, 1-month, and 2-month schedule. RTS,S/AS01B was given as either three full doses or with a fractional (1/5th) third dose. Efficacy was assessed by controlled human malaria infection (CHMI). Safety and immunogenicity of the vaccine regimen was also assessed. Forty-one malaria-naive adults received RTS,S/AS01B at 0, 4 and 8 weeks, either alone (Groups 1 and 2) or with ChAd63 ME-TRAP at week 0, and modified vaccinia Ankara (MVA) ME-TRAP at weeks 4 and 8 (Groups 3 and 4). Groups 2 and 4 received a fractional (1/5th) dose of RTS,S/AS01B at week 8. CHMI was delivered by mosquito bite 11 weeks after first vaccination. Vaccine efficacy was 6/8 (75%), 8/9 (88.9%), 6/10 (60%), and 5/9 (55.6%) of subjects in Groups 1, 2, 3, and 4, respectively. Immunological analysis indicated significant reductions in anti-circumsporozoite protein antibodies and TRAP-specific T cells at CHMI in the combination vaccine groups. This reduced immunogenicity was only observed after concomitant administration of the third dose of RTS,S/AS01B with the second dose of MVA ME-TRAP. The second dose of the MVA vector with a four-week interval caused significantly higher anti-vector immunity than the first and may have been the cause of immunological interference. Co-administration of ChAd63/MVA ME-TRAP with RTS,S/AS01B led to reduced immunogenicity and efficacy, indicating the need for evaluation of alternative schedules or immunization sites in attempts to generate optimal efficacy.

## Malaria: Efficacy of a multi-stage vaccination for Malaria

A number of Malaria vaccine candidates are currently under consideration. Targeting of multiple stages in the life cycle of *Plasmodium falciparum*, the causative agent of Malaria may increase vaccine efficacy. Clinical studies of such combinatorial vaccine approaches are required. Here Adrian Hill and colleagues at the University of Oxford report on their phase I/IIa study where they examine the effects of multiple combinational vaccine approaches that target the multiple stages of the parasite life cycle in a controlled human malaria infection model, assessing the efficacy and safety of these approaches in human volunteers prior to infection. Reduced immune responses and protection from Malaria infection was observed with co-administration of ME-TRAP containing vaccines. This study highlights a need to fully evaluate vaccination approaches to established more efficacious immunization protocols for Malaria and the potential of multi-stage approaches.

## Introduction

Although the incidence of malaria has decreased globally since 2000, it remains a leading cause of mortality. An estimated 3.2 billion people remain at risk of disease, and approximately 445,000 deaths were attributed to malaria in 2016.^1^ No licensed malaria vaccine is available, although several candidates are in development, at stages ranging from demonstrated efficacy in controlled human malaria infection (CHMI) studies,^2–5^ to completion of phase 3 efficacy testing and positive European Medicines Agency scientific opinion.^6,7^ A strategy for increasing vaccine efficacy (VE) is combining antigenically distinct vaccines, targeting different stages of the parasite life cycle, into a single regimen. There are strong arguments that combining vaccines targeting different stages of the parasite life cycle into one regimen could increase VE.^8–11^ Different vaccine platforms exert efficacy against malaria through differing immune mechanisms,^2–5^ and an additional benefit of combining vaccine types is induction of both humoral and cellular immune responses to potentially increase efficacy. Based on supportive pre-clinical findings,^12–14^ we previously reported a study demonstrating high VE (as defined by sterile protection (SP) of subjects) against CHMI (14/17 subjects protected; VE 82.4% (95% confidence interval (CI): 64–100)) in healthy, malaria-naive adults with an estimated sustained sterile efficacy of 72% observed in a subset of subjects who underwent re-challenge at 6 months.^15^ Subjects received a vaccination schedule consisting of three standard doses of the sporozoite stage subunit vaccine RTS,S/AS01B, in addition to the heterologous prime-boost viral vector vaccine regimen of ChAd63-modified vaccinia Ankara (ChAd63-MVA) multiple-epitope thrombospondin-related adhesion protein (ME-TRAP), which targets the liver stage of infection. This study was notable, not just because it demonstrated high VE, but also in that it combined two distinct vaccine types: the first (RTS,S) induces high-titer antibodies to the circumsporozoite protein (CSP) and another inducing potent T cell responses to TRAP using viral vectors (ChAd63-MVA ME-TRAP). Although the efficacy observed in the combination group was higher than in the comparator group that received three standard doses of RTS,S alone (12/16 subjects protected; VE 75% (95% CI: 54–96) estimated sustained VE at 6 months of 62.5%), the number of subjects in the study was small, and the difference in efficacy between the groups, or estimated sustained efficacy at re-challenge, was not statistically significant. The need for further evaluation of this approach was apparent. Furthermore, in this study, the RTS,S and viral vector vaccines were given separately at staggered time points, with a minimum interval of 2 weeks between each dose, resulting in a five-dose vaccination regimen, over a course of 10 weeks. Cumulative number of doses is a significant cost and logistic consideration for a vaccine regimen to be deployable in malaria endemic countries. Ideally, a malaria vaccine would be deliverable concurrently within the Expanded Program of Immunizations (EPI) such as the three-dose diphtheria, pertussis, and tetanus–hepatitis B virus vaccine.^16,17^

In 1997, during the first CHMI trial of RTS,S, reactogenicity concerns after the second dose of vaccine led to a reduction in the third dose in two of the study groups. One group received a regimen consisting of two standard doses of RTS,S/AS02 at 0 and 1 month, and a third dose at month 7 which was 1/5th of the standard dose. Following CHMI, 6/7 subjects remained protected, resulting in a VE of 86% (95% CI: 0.02–0.88, *P* < 0.005).^5^ The beneficial effect of a fractional third dose on VE was further demonstrated in a recent CHMI trial, in which subjects received a 0-month, 1-month, and 7-month regimen consisting of two standard doses of RTS,S/AS01B followed by a fractional (1/5th) third dose.^18^ Following CHMI, 26/30 subjects were protected against malaria (VE 86.7% (95% CI: 66.8–94.6)) compared with 10/16 in the standard 0-month, 1-month, 2-month full-dose group (VE 62.5 (95% CI: 29.4–80.1); log-rank *P* = 0.040).

In this phase I/IIa, open-label, CHMI study, we assessed whether the efficacy of a standard three-dose regimen RTS,S/AS01B could be improved by the concurrent, same site administration of ChAd63 and MVA viral vectors expressing ME-TRAP. Safety and immunogenicity of this novel schedule were also assessed. In addition, we assessed for the first time whether the efficacy of a standard three-dose regimen of RTS,S/AS01B could be improved by a regimen consisting of two standard doses followed by a fractional (1/5th) third dose given on a 0-month, 1-month, and 2-month schedule, either alone or given concurrently with viral vectors expressing ME-TRAP.

## Results

### Study participants

Seventy-four subjects were screened for eligibility and 45 subjects were identified as eligible at enrolment (Supplementary Figure SF1). Ten subjects each were allocated to Groups 1, 2, and 3 to receive R-R-R, R-R-r, or RA-RM-RM, respectively. Eleven subjects were allocated to Group 4 to receive RA-RM-rM. Group allocation numbers were lower than the planned 12 per group at CHMI, as a result of consent withdrawals and ineligibility at the time of first vaccination. Four subjects were allocated to Group 5 as unvaccinated infectivity controls. Vaccinations took place between 5 January 2015 and 27 February 2015. CHMI was performed on 23 March 2015 and 24 March 2015.

### Safety

The majority of adverse events (AEs) following vaccination in all vaccine groups were mild in severity and self-limiting. There were no serious AEs (SAEs) related to vaccination or suspected unexpected serious adverse reactions (SUSARs). Commonly reported AEs following vaccination were vaccine site pain, feverishness, fatigue, malaise, headache, and myalgia. There were no significant differences in the rates of occurrence of grade 3 (severe) solicited or unsolicited AEs, between the RTS,S/AS01B-alone groups (1 and 2) and the RTS,S/AS01B plus viral vectors groups (3 and 4) (Supplementary Table S4). Tabulations of AEs can be found in the Supplementary Tables S1–S5.

### Protective efficacy against CHMI

**Fig. 1:**
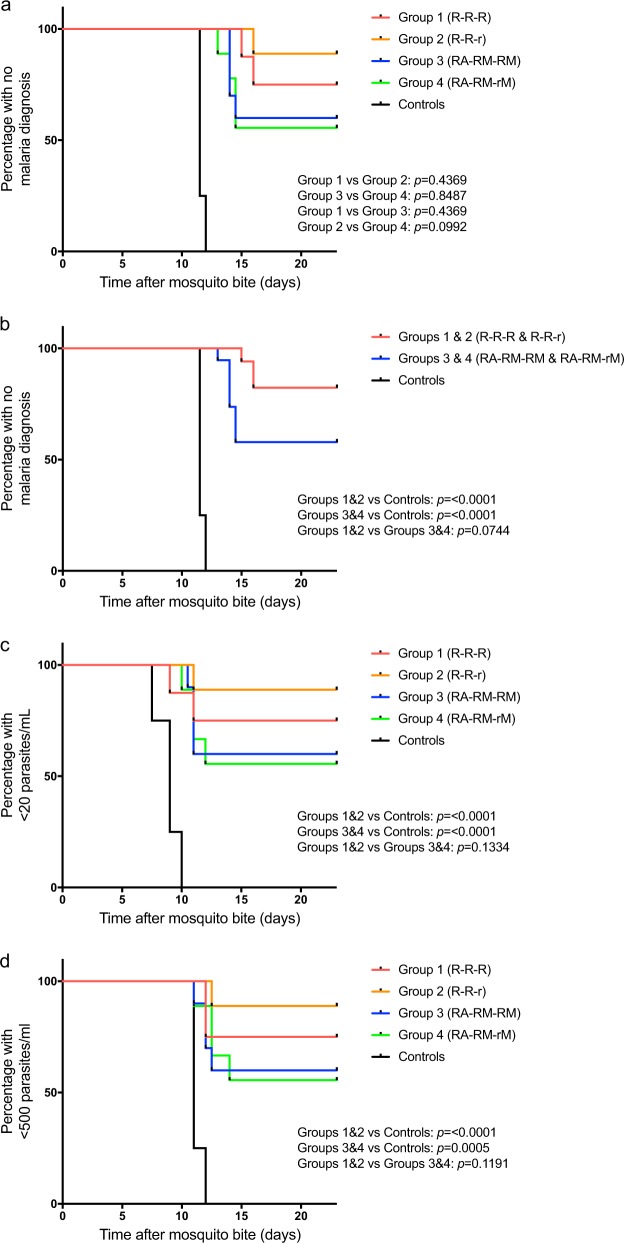
Efficacy of concomitant administration of RTS,S/AS01B with ChAd63-MVA ME-TRAP and RTS,S/AS01B immunization alone following *Plasmodium falciparum* 3D7 sporozoite challenge. Kaplan–Meier survival analyses. Log-rank test for significance. **a** Kaplan–Meier survival analysis of time to treatment following CHMI in individual groups. Mean time to diagnosis was 11.6 (±0.22) days for unvaccinated controls. All vaccine recipients were undiagnosed by day 23 after CHMI, or were diagnosed after the control mean ± 2 SD. **b** Kaplan–Meier survival analysis of time to treatment following CHMI in pooled vaccine groups. Vaccine recipients who had received a schedule consisting of RTS,S/AS01B alone (Groups 1 and 2) were pooled, and vaccine recipients who had received a schedule consisting of RTS,S/AS01B with viral vectors expressing ME-TRAP (Groups 3 and 4) were pooled. **c** Kaplan–Meier survival analysis of time to first sample with >20 parasites/mL detected by quantitative polymerase chain reaction (qPCR). Mean time to this endpoint was 8.9 (±0.89) days for unvaccinated controls. Seven out of eight subjects in Group 1 (87.5%), 9/9 (100%) subjects in Group 2, 9/10 (90%) subjects in Group 3, and 8/9 (88.9%) subjects in Group 4 did not reach this endpoint, or did so after the control mean ± 2 SD. **d** Kaplan–Meier survival analysis of time to first sample with >500 parasites/mL detected by qPCR. Mean time to this endpoint was 11.25 (±0.43) days for unvaccinated controls. Six out of eight subjects in Group 1 (75%), 9/9 (100%) subjects in Group 2, 7/10 (70%) subjects in Group 3, and 8/9 (88.9%) did not reach this endpoint, or did so after the control mean ± 2 SD. R-R-R, three standard doses (50 μg) of RTS,S/AS01B delivered at 4-week intervals; R-R-r, two standard doses (50 μg) of RTS,S/AS01B followed by a fractional third dose (10 μg). Vaccines delivered at 4-week intervals; RA-RM-RM, three standard doses (50 μg) of RTS,S/AS01B delivered at 4-week intervals with concomitant administration of Chimpanzee adenovirus 63 (ChAd63) expressing multiple-epitope thrombospondin-related adhesion protein (ME-TRAP) at week 0, and modified vaccinia Ankara (MVA) expressing ME-TRAP at weeks 4 and 8; RA-RM-rM, two standard doses (50 μg) of RTS,S/AS01B followed by a fractional third dose (10 μg) with concomitant administration of ChAd63 ME-TRAP at week 0, and MVA ME-TRAP at weeks 4 and 8

Forty subjects underwent CHMI and completed follow-up. By day (D) 23 following CHMI, 6/8 subjects in Group 1 were protected (VE 75% (95% CI: 31.5–93.1)); in Group 2, 8/9 subjects were protected (VE 88.9% (95% CI 43.3–98.4)); in Group 3, 6/10 subjects were protected (VE 60% (95% CI: 25.3–82.7)); in Group 4, 5/9 subjects were protected (VE 55.6% (95% CI: 20.4–80.5)) (Fig. 1a). All four unvaccinated controls in Group 5 were diagnosed with malaria. Pooling the outcome of subjects in Groups 1 and 2, who had received RTS,S/AS01B alone, 14/17 subjects were protected (82.4% (95% CI: 54.7–93.9)) compared with 11/19 subjects who had received RTS,S/AS01B with viral vectors in Groups 3 and 4 (VE 57.9% (95% CI: 33.2–76.3); *P* = 0.074) (Fig. 1b). Mean time to diagnosis was 11.6 days (range 11.5–12, SD = 0.22 days) in Group 5, while mean time to diagnosis was 15.5, 16.5, 14.1, and 14 days in Groups 1, 2, 3, and 4 respectively. Analysis of time to parasitemia measured by quantitative polymerase chain reaction (qPCR) showed significant difference in time to parasitemia for all vaccine groups compared with controls, using thresholds of either 20 or 500 parasites per milliliter of blood (Fig. 1c, d and Supplementary Table S6).

### T cell immunogenicity

T cell responses to vaccine antigens were measured by interferon γ (IFNγ) enzyme-linked immunosorbent spot (ELISPOT) assay (Supplementary Figure SF2). Responses at baseline and after vaccination were assessed to CSP in all groups and to ME-TRAP in Groups 3 and 4 only. CSP-specific T cell frequencies are described in the supplementary information (Supplementary Figure SF2A). T cell responses to ME-TRAP peaked 1 week after the first dose of MVA at D35 (Supplementary Figure SF2B) at a median of 2531 SFC (interquartile range (IQR): 1949–4042). This high level of cellular immunogenicity is comparable to what we previously observed after an 8-week prime-boost interval with ChAd63 and MVA ME-TRAP (2436 SFC, IQR: 1064–3862, Supplementary Figure 2C).^2^ Administration of a second dose of MVA induced a re-boosting of T cell responses but to a significantly lower median magnitude than after the first MVA dose (2531 SFC, IQR: 1949–4042 after first MVA compared with 901 SFC, IQR: 509–1910, *P* = 0.04, one-way analysis of variance).

Flow cytometry with intracellular cytokine staining (ICS) demonstrated that cytokine responses to CSP were predominantly from CD4^+^ T cells and were not significantly different between groups either at D42 or C-1 (Supplementary Figure SF3A). ICS responses to TRAP were assessed in Groups 3 and 4 only and comprised expression of cytokines from similar proportions of CD4^+^ and CD8^+^ T cells (Supplementary Figure SF3B). CD8^+^ T cell responses to TRAP were dominated by cells expressing IFNγ either alone or in combination with other cytokines. Responses did not change substantially between post Ad and post first MVA, but the second dose of MVA substantially reduced the proportion of monofunctional cells expressing IFNγ (D63 and C-1, *P* = 0.007 two-tailed *t* test), (Supplementary Figure SF4), a population that we have previously shown to be associated with vaccine-induced protection against malaria.^2^

### Humoral Immunogenicity of RTS,S/AS01B co-administered with ChAd63-MVA ME-TRAP

**Fig. 2:**
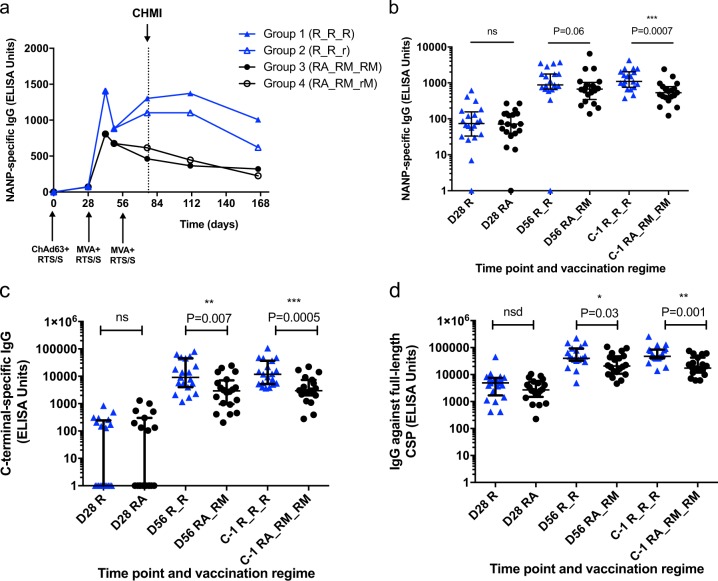
Humoral immunogenicity to CSP. **a** Median NANP IgG time courses. **b** NANP IgG responses after first RTS,S vaccination (day 28, D28), after second (day 56, D56), and third (C-1) RTS,S vaccinations, for volunteers receiving RTS,S/AS01B alone or RTS,S/AS01B and viral vectors co-administered. Two-tailed Mann–Whitney analyses. A trend to higher NANP IgG in RTS,S-alone group was observed after second vaccination *P* = 0.06 and significantly higher titers after third vaccination *P* = 0.0007 (***). **c** C-terminal IgG responses after each RTS,S vaccination. Significantly higher titers in RTS,S-only group after second and third vaccinations, two-tailed Mann–Whitney, 0.007 (**) and 0.0005 (***), respectively. D, IgG responses against full-length CSP. Significantly higher titers in RTS,S-only group after second and third vaccinations, two-tailed Mann–Whitney *P* = 0.03 (*) and 0.001 (**), respectively; Bars represent medians with interquartile range (IQR); n.s., not statistically significant

Antibody (Ab) responses induced by RTS,S vaccination were measured by enzyme-linked immunosorbent assay (ELISA) against the NANP repeats, C-terminal regions of CSP and the full-length CSP protein (Fig. 2a–d). NANP IgG responses peaked at D42 in all groups, declined by D56 and increased again following the third vaccination in the groups receiving RTS,S alone (Groups 1 and 2) but not in the RTS,S with viral vectors groups (Groups 3 and 4) (Fig. 2a). There was no significant difference in NANP IgG titers between Groups 1 and 2 pooled compared with Groups 3 and 4 pooled after first vaccination (Fig. 2b), a trend to higher titers in Groups 1 and 2 pooled after second vaccination (*P* = 0.06) and significantly higher titers in this group after third vaccination (*P* = 0.0007). C-terminal IgG titers were comparable between groups after the initial vaccination but were significantly lower in the RTS,S with viral vectors group after the second and third vaccinations (Fig. 2c *P* = 0.007 and 0.0005, respectively). Similarly, Ab responses against full-length CS protein were not significantly different between groups at D28, but were significantly higher in the RTS,S-only group at D56 and C-1 (Fig. 2d, *P* = 0.03, *P* = 0.001). There were no significant differences in anti-CSP IgG titers at C-1 when comparing the groups that received the fractional third dose of RTS,S with those that received the full dose (*P* = 0.905, *P* = 0.842 in comparing Groups 1 with Group 2, and Groups 3 with 4, respectively)

### Avidity of IgG responses to CSP and total IgG responses to TRAP

**Fig. 3:**
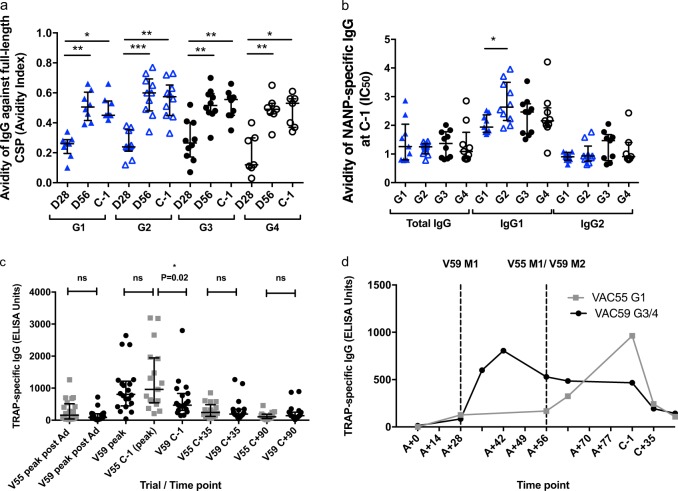
Avidity of IgG responses to CSP and total IgG responses to TRAP. **a** Avidity of total IgG against full-length CSP in each group at D28, D56, and C-1. Friedman test with Dunn’s correction for comparison of time points within each group *P* value = 0.0009, <0.0001, <0.0001, and 0.003 for G1, 2, 3, and 4, respectively. No significant difference between groups at any time point, Kruskal–Wallis analysis with Dunn’s correction. **b** Avidity of total IgG, IgG1, and IgG3 against NANP for G1, 2, 3, and 4. No significant differences between groups for total IgG or IgG2 avidity. Significantly higher IgG1 avidity in G2 compared with G1, two-tailed Mann–Whitney, *P* value = 0.01 (*), no significant differences between other groups or isotypes. **c** TRAP-specific IgG responses. RTS,S co-administered with vectors (VAC59 G3/4, black circles), and RTS,S and vectors administered at a 2-week interval (VAC55 G1, gray squares). No significant difference between trials at peak post ChAd vaccination (21 days after ChAd), 35 days post challenge (C + 35), or 90 days post challenge (C + 90) and peak post MVA vaccinations compared between trials, two-tailed Mann–Whitney. No significant difference between peak post MVA in VAC55 (C-1) and peak post first MVA in VAC59 (D42), significantly lower TRAP IgG titers at peak post second MVA in VAC59 (C-1), two-tailed Mann–Whitney *P* = 0.02 (*), respectively. **d** Median TRAP IgG time courses in VAC55 (gray squares) and VAC59 (black circles). R, full-dose RTS,S/AS01B; A, ChAd63 ME-TRAP; M, MVA ME-TRAP; r ractional dose RTS,S/AS01B; CHMI, controlled human malaria infection; C-1, day before CHMI; SFCs, spot-forming cells per million PBMC; n.s., not statistically significant; Bars represent medians with IQR. *= *P* ≤ 0.05; **= *P* ≤ 0.01; ***= *P* ≤ 0.001

Differences in the quality of the Ab response in each group were compared using a sodium thiocyanate displacement ELISA to measure avidity of total IgG against full-length CSP at D28, D56, and C-1 (Fig. 3a), and of total IgG, IgG1, and IgG2 against NANP at C-1 (Fig. 3b). Avidity of CSP-specific IgG increased significantly after the second vaccination and was not further increased by a third dose in any group. There were no significant differences in CSP IgG avidity between groups at any time point. There were no significant differences in the avidity of NANP-specific total IgG, or IgG2 between groups at C-1. The avidity of NANP-specific IgG1 was significantly higher in Group 2 compared with Group 1 directly (Mann–Whitney analysis, *P* = 0.01), but there were no significant differences in a comparison of all groups (Kruskal–Wallis with Dunn’s correction *P* = 0.0837). Total IgG responses to TRAP are described in Supplementary Information (Fig. 3c, d).

### Anti-vector Ab responses

**Fig. 4:**
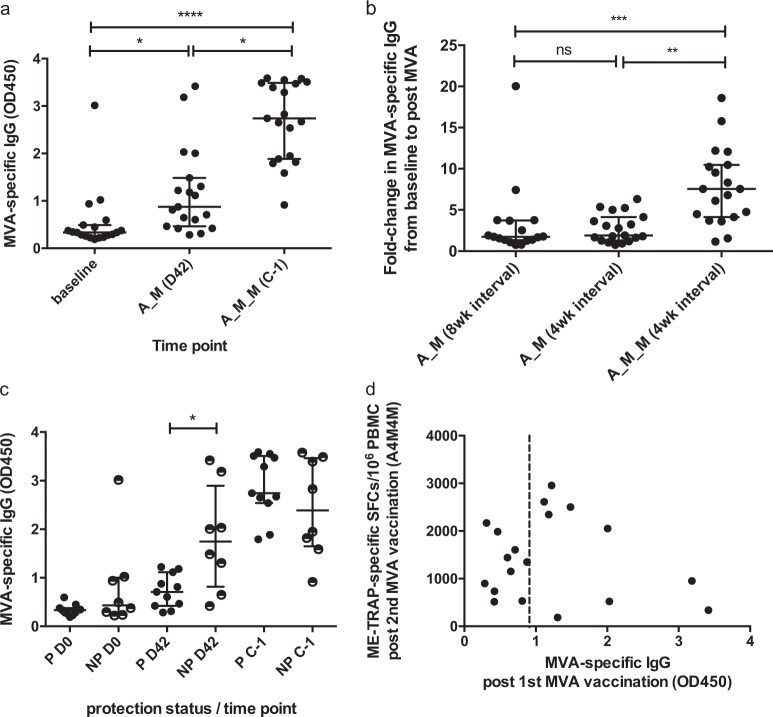
Anti-vector antibody responses. **a** Total IgG against MVA at baseline (D0), after first MVA vaccination (D42) and after second MVA vaccination (C-1) for G3/4. Kruskal–Wallis with Dunn’s correction, *P < 0.05, ****P < 0.0001. **b** Fold change in anti-MVA antibody from baseline to peak post MVA in study VAC055^15^ (MVA administered 8 weeks after ChAd63, A_M), peak post first MVA in VAC059 G3/4 (D42, A4_M) and peak post second MVA in VAC059 G3/4 (C-1, A_M_M), Kruskal–Wallis with Dunn’s correction *P* = 0.002 (***). **c** Anti-MVA IgG responses at baseline, post first and second MVA in volunteers protected (P) and non-protected (NP) after CHMI. Two-tailed Mann–Whitney comparison between groups at each time point, *P = 0.02 at D42. **d** Relationship between anti-MVA antibodies at peak post first MVA (D42) and ME-TRAP-specific T cell responses post second MVA at C-1 in VAC059 G3/4, Spearman’s *r* = −0.63, *P* value = 0.07. Bars represent medians with IQR

Antibody responses to MVA were measured using the WR113/D8L protein from MVA^19^ in Groups 3 and 4 by total IgG ELISA at baseline, and after each MVA vaccination (D42, D76/C-1) (Fig. 4a). At baseline, two volunteers were borderline positive and one volunteer was strongly positive for anti-MVA antibodies, although none had received smallpox vaccination. Anti-MVA IgG titers significantly increased after the first MVA vaccination and again after the second vaccination, after which all volunteers were seropositive (Kruskal–Wallis analysis with Dunn’s correction *P* < 0.0001). The increase in anti-vector antibodies after initial MVA vaccination at 4 weeks was comparable to that induced by a single MVA given at 8 weeks in a previous trial (Fig. 4b). However, the fold change in anti-MVA titers was significantly higher after two MVA doses (Kruskal–Wallis analysis with Dunn’s correction *P* = 0.0002). Anti-MVA titers at baseline and C-1 were comparable between protected and non-protected volunteers, but titers were significantly higher in non-protected volunteers after the initial MVA vaccination (Fig. 4c, *P* = 0.02). There was a trend towards a negative association between anti-MVA IgG after the initial MVA (D42) and TRAP-specific T cell responses after the second MVA (C-1) (Fig. 4d, *r* = −0.6333, *P* = 0.08), but no association between anti-MVA antibodies at D42 and TRAP-specific T cell responses at D42 (Supplementary Figure SF5A) or between anti-MVA antibodies at C-1 and TRAP-specific T cells at C-1 after a single MVA administered at 8 weeks (Supplementary Figure SF5B). However, some of the lowest C-1 TRAP T cell responses were observed in individuals with high titers of anti-MVA IgG at D42.

### Potential additive effect of TRAP-specific T cells to efficacy in co-administration groups

**Fig. 5:**
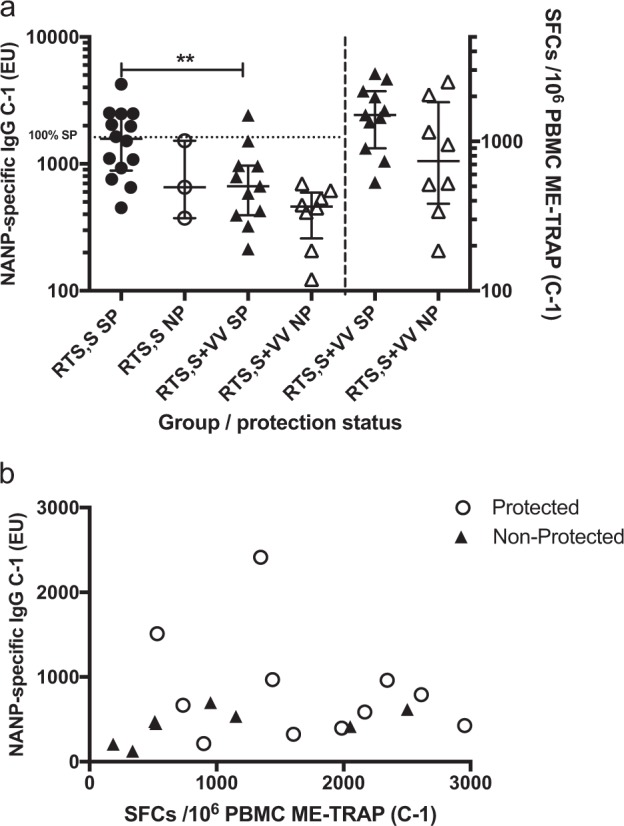
**a** CSP-specific IgG titers and TRAP-specific ELISPOT responses at C-1, stratified by vaccine regime and CHMI outcome. CSP-specific IgG titers (left *y*-axis) and TRAP-specific ELISPOT responses (right *y*-axis) at C-1, stratified by vaccine regime and CHMI outcome. **P = 0.009, two-tailed Mann–Whitney test. VV, viral vectors. Bars represent medians with IQR. **b** ELISPOT responses and ELISA titers for each individual participant with protection status after CHMI indicated. Circles indicate participants sterilely protected after CHMI and triangles non-protected participants

In the groups that received RTS,S alone (Groups 1 and 2), SP was achieved with CSP titers as low as 451 ELISA units (EU), with 100% of volunteers protected above 1600 EU. A statistically significant reduction in CSP IgG titer was observed between groups that received RTS,S in combination with viral vectors as compared to groups that received RTS,S alone (*p* = 0.009, two-tailed Mann–Whitney test, Fig. 5a, left-hand axis). Median NANP-specific IgG titers at C-1 in the combination group with SP (659 EU, IQR: 374–1520) were comparable to non-protected (NP) volunteers in the RTS,S-alone group (667, IQR: 394–968) and were not significantly different to median CSP IgG titers in non-protected volunteers receiving RTS,S with viral vectors (462 EU, IQR: 259–595, *P* = 0.15, Mann–Whitney test). Interestingly, median ELISPOT responses were twice as high in SP volunteers in the combination group (median 1505 SFC, IQR: 898–2167, Fig. 5, right-hand axis) compared with NP (median 738, I:QR 382–1827), although differences were not statistically significant (*P* = 0.09, Mann–Whitney test). Individual ELISPOT and Ab titers with protection status indicated are presented in Fig. 5b.

## Discussion

This is the second study to combine RTS,S and the viral vectors ChAd63 and MVA encoding ME-TRAP in the same regimen,^15^ but the first in which viral vectors have been concomitantly administered with RTS,S. It is the third study to evaluate an RTS,S^5,18^ regimen that incorporates a fractional third dose, but the first with it administered in a 0-month, 1-month, and 2-month schedule. We have shown that administering these vaccines concomitantly is safe, with no SUSARs, and no vaccine-related SAEs. As expected, a higher frequency of AEs was observed in the groups that received the viral vectors with RTS,S, but the majority of AEs were mild, and all were self-limiting.

In this study, we have again observed a high level of VE in the groups that received only RTS,S either at three full standard doses or with a reduced third dose. However, in the groups that received viral vectors and RTS,S, observed VE was lower than in the RTS,S-alone groups. Although no comparisons of VE between vaccinated groups are statistically significant, they suggest that concomitant administration of these vaccines, according to the schedules and immunization routes used in this trial, may reduce Ab responses to CSP and negatively impact VE.

As with previous CHMI studies of RTS,S/AS01B,^3,15,20^ anti-CSP Ab titers in this study were significantly higher in protected subjects immediately prior to CHMI than in non-protected subjects. In this study, the third dose of RTS,S appears to be ineffective at boosting to levels greater than those after the second dose, and in the subjects receiving RTS,S with viral vectors the third dose of RTS,S was ineffective at even maintaining anti-CSP Ab titers. As a result, anti-CSP Ab titers were significantly lower on the day preceding challenge (C-1) in the RTS,S plus viral vectors groups than in the RTS,S-alone groups.

Prior studies of RTS,S and viral vector combination regimens have administered RTS,S at intervals of at least 2 weeks from viral vectors.^15,21^ In the current study, subjects in Groups 3 and 4 received a combinations of antigens that included the NANP repeats and T cell epitopes of CSP, and the hepatitis B surface antigen in addition to multiple antigens on the surface of either the ChAd63 or MVA vectors. With simultaneous exposure to multiple antigens, there is a potential for each to interfere with immune responses to the others, and in this study the interval between administration of MVA doses was also reduced, which may have also contributed to intereference. Studies of non-malaria vaccines show that immune interference is complex and may result in enhancement or depression of responses to one or more antigens^22^ and therefore may alter the resulting efficacy. In this trial Groups 3 and 4 received a viral vector at the same immunization site as RTS,S in addition to the potent adjuvant AS01B. This appears to have enhanced immune responses to the MVA vector, but not T cells to ME-TRAP expressed by the MVA insert. The second dose of MVA co-administered with RTS,S/AS01B at the third immunization time point led to no boosting of the Ab response to CSP. Notably, we observed a profound boosting effect of the second vaccination on anti-CSP Ab titers in all the trial groups, with strong Ab responses to the MVA vector protein induced by the first MVA at 4 weeks suggesting that this anti-vector immunity might have contributed to lack of boosting of CSP-specific antibodies after the third dose. This striking immunological interference was surprising as pre-clinical murine studies found no detrimental impact of same site administration on immunogenicity or efficacy of the vaccine components (Collins et al., unpublished).

Vaccine-induced TRAP-specific CD8^+^ T cells have previously been shown to correlate with protection against CHMI.^2^ In this study, we observed protection in some subjects with very low anti-CSP Ab titers, but substantial TRAP-specific T cell responses. This supports, but does not prove, the hypothesis that where titers of CSP antibodies are suboptimal for protection, TRAP-specific T cells induced by vaccination could potentially add a substantial element of protection by eliminating a reduced number of infected hepatocytes following the anti-sporozoite effect of CSP antibodies. The failure of the third dose of RTS,S to boost anti-CSP antibodies when administered with viral vectors in this study, however, is clearly concerning, and would negate any potential additional beneficial effect of TRAP-specific T cells on efficacy. The numbers of non-protected subjects, or subjects with low anti-CSP antibodies in the RTS,S-alone group, was too small to draw any meaningful statistical comparisons with subjects in the combination groups with similarly low anti-CSP Ab titers. Further work would be necessary to determine whether the negative effect of concomitant administration can be overcome and to identify whether vaccines inducing TRAP-specific T cells could contribute to efficacy in combination with anti-CSP Ab-inducing vaccines when administered simultaneously. Alternative solutions include concomitant administration but at different vaccination sites, administering only a single dose of MVA ME-TRAP, or adjusting the vaccine dose or formulation. Other options include separation of the administration time points of the anti-sporozoite-stage and anti-liver-stage vectors, based on the durable high efficacy of this approach which we reported previously.^15^ The RTS,S phase 3 trial showed highest immunogenicity and efficacy of this vaccine in children aged 5–17 months compared to 6–12 week olds, leading to the WHO recommendation for pilot implementation in the older age group. In addition, anti-CSP IgG titers are a surrogate of protection, with reduced efficacy and durability also observed in the younger age group.^23^ In contrast, recent phase Ib data on ME-TRAP vector administration in Gambian and Burkinabe infants^24,25^ showed optimal T cell immunogenicity in 2–4-month-old infants even when the vectored vaccines were co-administered with standard EPI vaccines. Hence, vaccination strategies aimed at exploiting the differences in immune responses in this younger age group should be explored.

In conclusion, no safety concerns arose from concomitant administration of ChAd63 and MVA viral vectors encoding ME-TRAP with RTS,S. Further work is required to evaluate the impact of concomitant administration, and the use of a fractional third dose of RTS,S on VE.

## Methods

### Participants

Recruitment and vaccination was conducted at four UK sites: Oxford, Southampton, London, and Guildford. The CHMI procedure was performed as previously described^26^ using five infectious bites from *P. falciparum* 3D7-strain-infected *Anopheles stephensi* mosquitoes at Imperial College, London. All subjects were infected with a single batch of infected mosquitoes supplied by the Department of Entomology, Walter Reed Army Institute of Research, Washington DC, USA. Healthy, malaria-naive males and non-pregnant females aged 18–45 years were invited to participate in the study. Volunteers gave written informed consent prior to participation. The study was conducted according to the principles of the Declaration of Helsinki and in accordance with Good Clinical Practice (GCP).

### Ethical and regulatory approval

Required approvals for the study were granted by the UK National Research Ethics Service, Committee South Central—Oxford A (Ref: 14/SC/0227), and the UK Medicines and Healthcare Products Regulatory Agency (Ref: 21584/0333/001-0001). The trial was registered with ClinicalTrials.gov (Ref: NCT02252640). The study was conducted according to all relevant guidelines and procedures. The Local Safety Committee provided safety oversight and GCP compliance was monitored by the Clinical Trials and Research Governance Team of the University of Oxford.

### Study design

This phase IIa, open-label, partially randomized challenge trial consisted of four vaccine cohorts (target *n* = 13) and an unvaccinated infectivity control group (*n* = 4). Vaccine regimens (Table 1) consisted of three doses of RTS,S/AS01_B_ alone, or concomitanty administered with ChAd63/MVA ME-TRAP. All vaccinations were administered intramuscularly (IM) into the deltoid region of the arm. For subjects in Groups 3 and 4, RTS,S/AS01B was administered first followed by viral vector vaccination at the same site no longer than 5 min after the RTS,S vaccination. All subjects underwent CHMI by mosquito bite at the same time (3 weeks after final vaccination for vaccinated subjects). Following CHMI, a diagnosis of blood stage malaria infection was made in subjects with symptoms suggestive of malaria and positive thick film microscopy, or qPCR result >500 parasites/mL if either thick film was negative, or symptoms were absent.^27^ Vaccinated subjects who had not developed blood stage malaria detectable by any assay by D23 after CHMI were deemed sterilely protected.

**Table 1:** Study design

Week	Group 1 (*n* = 13)R-R-R	Group 2 (*n* = 13)R-R-r	Group 3 (*n* = 13)RA-RM-RM	Group 4 (*n* = 13)RA-RM-rM	Group 5 (*n* = 4)Control
0	RTS,S/AS01_B_ (Standard dose)	RTS,S/AS01_B_ (Standard dose)	RTS,S/AS01_B_ (Standard dose) and ChAd63 ME-TRAP	RTS,S/AS01_B_ (Standard dose) and ChAd63 ME-TRAP	
4	RTS,S/AS01_B_ (Standard dose)	RTS,S/AS01_B_ (Standard dose)	RTS,S/AS01_B_ (Standard dose) and MVA ME-TRAP	RTS,S/AS01_B_ (Standard dose) and MVA ME-TRAP	
8	RTS,S/AS01_B_ (Standard dose)	RTS,S/AS01_B_ (1/5 Standard dose)	RTS,S/AS01_B_ (Standard dose) and MVA ME-TRAP	RTS,S/AS01_B_ (1/5 Standard dose) and MVA ME-TRAP	
11	CHMI (*n* = 12)	CHMI (*n* = 12)	CHMI (*n* = 12)	CHMI (*n* = 12)	CHMI

Group 1 received 3 standard doses (50 µg) of RTS,S/AS01B (R-R-R); Group 2 received two standard doses of RTS,S/AS01B followed by a third fractional dose of RTS,S/AS01B at 1/5th of the standard dose (R-R-r); Group 3 received three standard doses of RTS,S/AS01B in addition to ChAd63 ME-TRAP 5 × 1010 virus particles (vp) at week 0 and MVA ME-TRAP 2 × 108 plaque-forming units (PFU) at weeks 4 and 8 (RA-RM-RM); Group 4 received two standard doses of RTS,S/AS01B followed by a third fractional dose of RTS,S/AS01B at 1/5th of the standard dose in addition to ChAd63 ME-TRAP 5 × 1010 vp at week 0 and MVA ME-TRAP 2 × 108 PFU at weeks 4 and 8 (RA-RM-rM). Group 5 (*n* = 4) received no vaccinations

Further details of the sample size, study sites, inclusion/exclusion criteria, the vaccines, randomization, clinical follow-up, safety monitoring, malaria treatment and diagnosis, immunological and molecular methods, and statistics are given in the Supplementary material.

### Previous presentations

Some of the data in this manuscript were previously presented orally at the American Society of Tropical Medicine and Hygiene annual meeting in October 2015, abstract 1277 http://www.abstractsonline.com/Plan/ViewAbstract.aspx?sKey=bdff3977-bc89-4514-a8af-8e9f04867120&cKey=f2cfd4b7-7966-474d-98ba-5ad1b5cca678&mKey=%7bAB652FDF-0111-45C7-A5E5-0BA9D4AF5E12%7d

## Electronic supplementary material

Supplementary Information

## Data Availability

The data that support the findings of this study are available from the corresponding author upon reasonable request.

## Electronic supplementary material

**Supplementary information** accompanies the paper on the *npj Vaccines* website (10.1038/s41541-018-0084-2).
