# Supplementary material for: Safety and efficacy of novel malaria vaccine regimens of RTS,S/AS01B alone, or with concomitant ChAd63-MVA-vectored vaccines expressing ME-TRAP
Source: NPJ Vaccines. 2018 Oct 9;3:49. doi: 10.1038/s41541-018-0084-2 (PMC6177476; doi:10.1038/s41541-018-0084-2)
Supplement: Supplementary file 1 — Supplementary Information [file 41541_2018_84_MOESM1_ESM.docx]

**Safety and Efficacy of Novel Malaria Vaccine Regimens of RTS,S/AS01B alone, or with Concomitantly Administered ChAd-MVA Vectored Vaccines Expressing ME-TRAP.**

Tommy Rampling^1^*^^^, Katie J. Ewer^1^^, Georgina Bowyer^1^, Nick J. Edwards^1^, Danny Wright^1^, Saranya Sridhar^1^, Ruth Payne^1^, Jonathan Powlson^1^, Carly Bliss^1^, Navin Venkatraman^1^, Ian D. Poulton^1^, Hans de Graaf^2^, Diane Gbesemete^2^, Amy Grobbelaar^1^, Huw Davies^3^, Rachel Roberts^1^, Brian Angus^1^, Karen Ivinson^4^, Rich Weltzin^4^, Bebi-Yassin Rajkumar^4^, Ulrike Wille-Reece^4^, Cynthia Lee^4^, Chris Ockenhouse^4^, Robert E. Sinden^5^, Stephen Gerry^6^, Alison M. Lawrie^1^, Johan Vekemans^7^, Danielle Morelle^7^, Marc Lievens^7^, Ripley W. Ballou^7^, David J. M. Lewis^8^, Graham S. Cooke^9^, Saul N. Faust^2^, Sarah Gilbert^1^, Adrian V.S Hill^1^.

Table of contents.

1. Supplementary Tables 5

1.1. Solicited adverse events by vaccine dose and group 5

1.2. Unsolicited AE Summary 8

1.3. Unsolicited Adverse Events Full Listing by Group & Vaccine 9

1.4. Solicited AE Comparison 18

1.5. Laboratory AEs 20

Table S7: CSP peptides sequences. 23

Table S8: CSP peptide pools. 23

Table S9: TRAP peptide sequences and pools. 25

Table S10: Antibodies used for flow cytometry. 25

2. Supplementary Figures and Results 26

2.1. Supplementary Figure SF1 26

Supplementary T cell immunogenicity 27

2.2. Supplementary Figure 2. 28

2.1. Supplementary Figure 3. 30

2.1. Supplementary Figure 4 31

Supplementary ELISA results 32

2.2. Supplementary Figure 5 33

Supplementary Figure 6 34

3. Supplementary Methods 35

3.1. Study Sites 35

3.2. Objectives 35

**3.2.1. PRIMARY OBJECTIVE 35**

**3.2.2. SECONDARY OBJECTIVES 36**

3.3. Sample Size 36

3.4. Inclusion/Exclusion Criteria 37

**3.4.1. Inclusion Criteria 37**

**3.4.2. Exclusion Criteria 38**

3.5. Vaccines 40

**3.5.1. RTS,S/AS01_B_ Vaccine 41**

**3.5.2. ChAd63-MVA ME-TRAP Vaccines 41**

**3.5.3. Vaccine administration 41**

3.6. Randomisation 41

3.7. Assessment of Safety 42

**3.7.1. Definitions 42**

3.8. Causality assessment 44

3.9. Malaria Diagnosis 44

3.10. *Ex-vivo* Interferon-γ (IFN-γ) Enzyme-Linked Immunosorbent Spot (ELISPOT) assays 45

3.11. Peptides for T cell Assays (Tables S6-S8) 45

3.12. Flow cytometry with Intracellular Cytokine Staining (ICS) 46

3.13. IgG ELISAs 47

3.14. Total IgG Enzyme Linked Immunosorbent Assay (ELISA) to TRAP 47

3.15. Total IgG Enzyme Linked Immunosorbent Assay (ELISA) to CS [10] 49

3.16. CSP-specific IgG Avidity ELISA 50

3.17. Anti-MVA IgG ELISA 50

3.18. Parasite Quantitative PCR (qPCR) 51

3.19. Statistical Analysis 51

4. References 51

# Supplementary Tables

## Solicited adverse events by vaccine dose and group

| **A: Vaccine 1 : Group 1&2 (n=20)** | | | |  | **Vaccine 1: Group 3&4 (n=21)** | | | |
| --- | --- | --- | --- | --- | --- | --- | --- | --- |
|  | **Mild (%)** | **Mod (%)** | **Sev (%)** |  |  | **Mild (%)** | **Mod (%)** | **Sev (%)** |
| Pain | 11 (55) | 7 (35) | 0 |  | Pain | 13 (61.9) | 7 (33.3) | 1 (4.8) |
| Warmth | 5 (25) | 0 | 0 |  | Warmth | 6 (28.6) | 4 (19) | 0 |
| Swelling | 1 (5) | 0 | 0 |  | Swelling | 1 (4.8) | 0 | 0 |
| Redness | 3 (15) | 3 (15) | 0 |  | Redness | 6 (28.6) | 0 | 0 |
| Itch | 1 (5) | 0 | 0 |  | Itch | 3 (14.3) | 0 | 0 |
| Fever | 0 | 0 | 0 |  | Fever | 3 (14.3) | 6 (28.6) | 1 (4.8) |
| Feverishness | 8 (40) | 1 (5) | 0 |  | Feverishness | 6 (28.6) | 7 (33.3) | 3 (14.3) |
| Myalgia | 9 (45) | 2 (10) | 0 |  | Myalgia | 9 (42.9) | 4 (19) | 1 (4.8) |
| Arthralgia | 0 | 1 (5) | 0 |  | Arthralgia | 10 (47.6) | 1 (4.8) | 1 (4.8) |
| Headache | 7 (35) | 2 (10) | 0 |  | Headache | 8 (38.1) | 4 (19) | 2 (9.5) |
| Fatigue | 10 (50) | 1 (5) | 1 (5) |  | Fatigue | 12 (57.1) | 3 (14.3) | 1 (4.8) |
| Nausea | 2 (10) | 0 | 0 |  | Nausea | 5 (23.8) | 1 (4.8) | 0 |
| Malaise | 6 (30) | 0 | 0 |  | Malaise | 10 (47.6) | 4 (19) | 1 (4.8) |

| **B: Vaccine 2: Group 1&2 (n=20)** | | | |  | **Vaccine 2: Group 3&4 (n=20)** | | | |
| --- | --- | --- | --- | --- | --- | --- | --- | --- |
|  | **Mild (%)** | **Mod (%)** | **Sev (%)** |  |  | **Mild (%)** | **Mod (%)** | **Sev (%)** |
| Pain | 10 (50) | 4 (20) | 1 (5) |  | Pain | 10 (50) | 9 (45) | 1 (5) |
| Warmth | 7 (35) | 1 (5) | 0 |  | Warmth | 7 (35) | 5 (25) | 1 (5) |
| Swelling | 0 | 0 | 0 |  | Swelling | 3 (15) | 2 (10) | 2 (10) |
| Redness | 2 (10) | 3 (15) | 1 (5) |  | Redness | 6 (30) | 3 (15) | 1 (5) |
| Itch | 2 (10) | 0 | 0 |  | Itch | 6 (30) | 1 (5) | 0 |
| Fever | 4 (20) | 3 (15) | 0 |  | Fever | 2 (10) | 2 (10) | 3 (15) |
| Feverishness | 6 (30) | 8 (40) | 1 (5) |  | Feverishness | 7 (35) | 9 (45) | 3 (15) |
| Myalgia | 12 (60) | 1 (5) | 1 (5) |  | Myalgia | 3 (15) | 10 (50) | 3 (15) |
| Arthralgia | 3 (15) | 3 (15) | 1 (5) |  | Arthralgia | 1 (5) | 8 (40) | 1 (5) |
| Headache | 7 (35) | 4 (20) | 1 (5) |  | Headache | 10 (50) | 7 (35) | 0 |
| Fatigue | 7 (35) | 6 (30) | 2 (10) |  | Fatigue | 6 (30) | 9 (45) | 3 (15) |
| Nausea | 6 (30) | 0 | 0 |  | Nausea | 10 (50) | 0 | 0 |
| Malaise | 7 (35) | 5 (25) | 3 (15) |  | Malaise | 8 (40) | 7 (35) | 2 (10) |

| **C: Vaccine 3: Group 1 (n=9)** | | | |  | **Vaccine 3: Group 2 (n=10)** | | | |
| --- | --- | --- | --- | --- | --- | --- | --- | --- |
|  | **Mild (%)** | **Mod (%)** | **Sev (%)** |  |  | **Mild (%)** | **Mod (%)** | **Sev (%)** |
| Pain | 8 (88.9) | 0 | 0 |  | Pain | 5 (50) | 1 (10) | 0 |
| Warmth | 4 (44.4) | 1 (11.1) | 0 |  | Warmth | 3 (30) | 0 | 0 |
| Swelling | 0 | 0 | 0 |  | Swelling | 1 (10) | 0 | 0 |
| Redness | 1 (11.1) | 1 (11.1) | 0 |  | Redness | 0 | 0 | 0 |
| Itch | 3 (33.3) | 0 | 0 |  | Itch | 0 | 0 | 0 |
| Fever | 8 (88.9) | 0 | 0 |  | Fever | 0 | 0 | 0 |
| Feverishness | 3 (33.3) | 1 (11.1) | 1 (11.1) |  | Feverishness | 0 | 0 | 0 |
| Myalgia | 4 (44.4) | 1 (11.1) | 1 (11.1) |  | Myalgia | 4 (40) | 0 | 0 |
| Arthralgia | 3 (33.3) | 1 (11.1) | 0 |  | Arthralgia | 2 (20) | 0 | 0 |
| Headache | 4 (44.4) | 0 | 0 |  | Headache | 3 (30) | 0 | 0 |
| Fatigue | 4 (44.4) | 2 (22.2) | 0 |  | Fatigue | 5 (50) | 0 | 0 |
| Nausea | 2 (22.2) | 0 | 0 |  | Nausea | 2 (20) | 0 | 0 |
| Malaise | 3 (33.3) | 2 (22.2) | 1 (11.1) |  | Malaise | 3 (30 | 0 | 0 |

| **Vaccine 3: Group 3 (n=10)** | | | |  | **Vaccine 3: Group 4 (n=10)** | | | |
| --- | --- | --- | --- | --- | --- | --- | --- | --- |
|  | **Mild (%)** | **Mod (%)** | **Sev (%)** |  |  | **Mild (%)** | **Mod (%)** | **Sev (%)** |
| Pain | 6 (60) | 3 (30) | 0 |  | Pain | 5 (50) | 2 (20) | 0 |
| Warmth | 6 (60) | 1 (10) | 1 (10) |  | Warmth | 3 (30) | 2 (20) | 0 |
| Swelling | 1 (10) | 0 | 1 (10) |  | Swelling | 0 | 2 (20) | 1 (10) |
| Redness | 4 (40) | 2 (20) | 0 |  | Redness | 5 (50) | 0 | 0 |
| Itch | 5 (50) | 0 | 0 |  | Itch | 1 (10) | 0 | 0 |
| Fever | 1 (10) | 1 (10) | 0 |  | Fever | 1 (10) | 0 | 0 |
| Feverishness | 2 (20) | 5 (50) | 0 |  | Feverishness | 3 (30) | 2 (20) | 0 |
| Myalgia | 6 (60) | 2 (20) | 1 (10) |  | Myalgia | 4 (40) | 1 (10) | 0 |
| Arthralgia | 3 (30) | 1 (10) | 1 (10) |  | Arthralgia | 3 (30) | 1 (10) | 0 |
| Headache | 4 (40) | 2 (20) | 0 |  | Headache | 3 (30) | 1 (10) | 0 |
| Fatigue | 7 (70) | 2 (20) | 0 |  | Fatigue | 5 (50) | 2 (20) | 0 |
| Nausea | 3 (30) | 0 | 0 |  | Nausea | 0 | 1 (10) | 0 |
| Malaise | 5 (50) | 2 (20) | 0 |  | Malaise | 1 (10) | 3 (30) | 0 |

**Table S1: Solicited adverse events reported in the 7 day period following each vaccination A: Vaccination 1. Group 1 & 2 subjects received a full dose of RTS,S/AS01_B_ only; Group 3 & 4 received a full dose of RTS,S/AS01_B_ with a concomitant dose of ChAd63 ME-TRAP B: Vaccination 2. Group 1 & 2 subjects received a full dose of RTS,S/AS01_B_ only; Group 3 & 4 received a full dose of RTS,S/AS01_B_ with a concomitant dose of MVA ME-TRAP. C: Vaccination 3. Group 1 subjects received a full dose of RTS,S/AS01_B_ only; Group 2 subjects received a fractional dose of RTS,S/AS01_B_ at 1/5^th^ standard dose only; Group 3 received a full dose of RTS,S/AS01_B_ with a concomitant dose of MVA ME-TRAP; Group 4 received a fractional dose of RTS,S/AS01_B_ at 1/5^th^ standard dose with a concomitant dose of MVA ME-TRAP**

## Unsolicited AE Summary

**A:**

|  | **1^st^ Vaccination** | | **2^nd^ Vaccination** | | **3^rd^ Vaccination** | |
| --- | --- | --- | --- | --- | --- | --- |
|  | **n (%)** | ***p*** | **n (%)** | ***p*** | **n (%)** | ***p*** |
| **Group 1 & 2** | 10 (50) | 0.0014 | 14 (70) | 0.2351 | 11 (57.9) | 0.5145 |
| **Group 3 & 4** | 20 (95.2) |  | 18 (90) |  | 14 (70) |  |
| **Group 1** |  |  |  |  | 4 (44.4) | 0.3698 |
| **Group 2** |  |  |  |  | 7 (70) |  |
| **Group 3** |  |  |  |  | 7 (70) | 1 |
| **Group 4** |  |  |  |  | 7 (70) |  |

**B:**

|  | **1^st^ Vaccination** | | **2^nd^ Vaccination** | | **3^rd^ Vaccination** | |
| --- | --- | --- | --- | --- | --- | --- |
|  | **n (%)** | ***p*** | **n (%)** | ***p*** | **n (%)** | ***p*** |
| **Group 1 & 2** | 10 (50) | 0.0203 | 11 (55) | 0.176 | 7 (36.8) | 0.1128 |
| **Group 3 & 4** | 18 (85.7) |  | 16 (80) |  | 13 (65) |  |
| **Group 1** |  |  |  |  | 3 (33.3) | 1 |
| **Group 2** |  |  |  |  | 4 (40) |  |
| **Group 3** |  |  |  |  | 7 (70) | 1 |
| **Group 4** |  |  |  |  | 6 (60) |  |

**Table S2: Unsolicited AE summary. A: Number of subjects with at least one report of unsolicited AE in the 28 day period post vaccination (Days 0-28). 2-tailed p value obtained using Fisher’s exact test.B: Number of subjects with at least one report of an unsolicited AE deemed possibly, probably or definitely related to vaccination in the 30 day period post vaccination (Days 0-28). 2-tailed p value obtained using Fisher’s exact test.**

## Unsolicited Adverse Events Full Listing by Group & Vaccine

**A: Groups 1 & 2 Vaccine 1**

| **PT code** | **Meddra PT** | **Day of onset (relative to vaccination)** | **Last day reported (relative to vaccination)** | **Max Severity** | **Causality** |
| --- | --- | --- | --- | --- | --- |
| 10003988 | Back pain | 0 | 0 | 1 | Possible |
| 10008531 | Chills | 0 | 1 | 1 | Probable |
| 10061428 | Decreased appetite | 0 | 0 | 1 | Probable |
| 10020568 | Hyperaesthesia | 1 | 1 | 1 | Possible |
| 10068319 | Oropharyngeal pain | 1 | 1 | 1 | Possible |
| 10000087 | Abdominal pain upper | 1 | 1 | 1 | Possible |
| 10018964 | Haemoptysis | 2 | 2 | 1 | Possible |
| 10000087 | Abdominal pain upper | 3 | 3 | 1 | Possible |
| 10068319 | Oropharyngeal pain | 6 | 7 | 1 | Possible |
| 10068319 | Oropharyngeal pain | 7 | 7 | 1 | Unlikely |
| 10016256 | Fatigue | 7 | 9 | 1 | Possible |
| 10068319 | Oropharyngeal pain | 9 | 9 | 1 | Unlikely |
| 10019211 | Headache | 9 | 10 | 1 | Unlikely |
| 10012735 | Diarrhoea | 10 | 10 | 2 | Unlikely |
| 10011224 | Cough | 10 | 11 | 2 | Unlikely |
| 10011224 | Cough | 13 | 15 | 1 | Unlikely |
| 10019211 | Headache | 13 | 14 | 1 | Unlikely |
| 10068319 | Oropharyngeal pain | 14 | 14 | 1 | Unlikely |
| 10028735 | Nasal congestion | 14 | 15 | 1 | Unlikely |
| 10068319 | Oropharyngeal pain | 15 | 17 | 1 | Unlikely |
| 10019211 | Headache | 13 | 15 | 2 | Unlikely |
| 10028813 | Nausea | 17 | 18 | 2 | Unlikely |
| 10019211 | Headache | 18 | 18 | 1 | Unlikely |
| 10068319 | Oropharyngeal pain | 17 | 20 | 2 | Unlikely |
| 10068319 | Oropharyngeal pain | 19 | 21 | 1 | Unlikely |
| 10011224 | Cough | 19 | 21 | 1 | Unlikely |
| 10019211 | Headache | 20 | 20 | 1 | Unlikely |
| 10013946 | Dyspepsia | 25 | 26 | 1 | Unlikely |
| 10028810 | Nasopharyngitis | 26 | 27 | 1 | Unlikely |
| 10019211 | Headache | 25 | 26 | 2 | Unlikely |
| 10019211 | Headache | 26 | 27 | 2 | Unlikely |
| 10028813 | Nausea | 27 | 27 | 2 | Unlikely |
| 10028411 | Myalgia | 27 | 27 | 2 | Unlikely |

**B: Groups 1 & 2 Vaccine 2**

| **PT code** | **Meddra PT** | **Day of onset (relative to vaccination)** | **Last day reported (relative to vaccination)** | **Max Severity** | **Causality** |
| --- | --- | --- | --- | --- | --- |
| 10008531 | Chills | 0 | 0 | 1 | Probable |
| 10043458 | Thirst | 1 | 1 | 3 | Possible |
| 10037844 | Rash | 1 | 1 | 1 | Possible |
| 10015150 | Erythema | 1 | 1 | 1 | Probable |
| 10008531 | Chills | 1 | 1 | 2 | Probable |
| 10010947 | Coordination abnormal | 2 | 2 | 1 | Possible |
| 10014020 | Ear pain | 2 | 2 | 1 | Possible |
| 10068319 | Oropharyngeal pain | 3 | 3 | 1 | Possible |
| 10011224 | Cough | 3 | 5 | 1 | Possible |
| 10037844 | Rash | 5 | 5 | 1 | Possible |
| 10009245 | Clavicle fracture | 6 | 6 | 1 | Possible |
| 10016256 | Fatigue | 8 | 10 | 2 | Possible |
| 10019211 | Headache | 8 | 10 | 2 | Possible |
| 10019211 | Headache | 8 | 9 | 1 | Possible |
| 10017553 | Furuncle | 11 | 20 | 1 | Unlikely |
| 10019211 | Headache | 12 | 12 | 1 | Unlikely |
| 10013946 | Dyspepsia | 13 | 13 | 1 | Unlikely |
| 10016256 | Fatigue | 13 | 13 | 2 | Possible |
| 10028813 | Nausea | 13 | 13 | 2 | Unlikely |
| 10003239 | Arthralgia | 13 | 15 | 1 | Unrelated |
| 10019211 | Headache | 14 | 14 | 1 | Unlikely |
| 10025482 | Malaise | 15 | 18 | 1 | Possible |
| 10011224 | Cough | 16 | 20 | 2 | Unlikely |
| 10024968 | Lower respiratory tract infection | 16 | 16 | 1 | Unlikely |
| 10019211 | Headache | 16 | 17 | 1 | Unlikely |
| 10024770 | Local swelling | 16 | 20 | 1 | Unlikely |
| 10028735 | Nasal congestion | 17 | 18 | 2 | Unlikely |
| 10067152 | Oral herpes | 17 | 19 | 1 | Unlikely |
| 10009245 | Clavicle fracture | 18 | 20 | 3 | Unrelated |
| 10012735 | Diarrhoea | 19 | 20 | 1 | Unlikely |
| 10029410 | Night sweats | 19 | 19 | 1 | Possible |
| 10041232 | Sneezing | 19 | 20 | 1 | Unlikely |
| 10000087 | Abdominal pain upper | 20 | 20 | 1 | Unlikely |
| 10019211 | Headache | 20 | 20 | 1 | Unlikely |
| 10039101 | Rhinorrhoea | 21 | 21 | 1 | Unlikely |
| 10036790 | Productive cough | 21 | 21 | 1 | Unlikely |

**C: Group 1 Vaccine 3**

| **PT code** | **Meddra PT** | **Day of onset (relative to vaccination)** | **Last day reported (relative to vaccination)** | **Max Severity** | **Causality** |
| --- | --- | --- | --- | --- | --- |
| 10012735 | Diarrhoea | 0 | 0 | 1 | Possible |
| 10011224 | Cough | 0 | 1 | 1 | Possible |
| 10039101 | Rhinorrhoea | 0 | 1 | 1 | Possible |
| 10016326 | Feeling cold | 1 | 1 | 2 | Possible |
| 10027599 | Migraine | 3 | 4 | 1 | Possible |
| 10012735 | Diarrhoea | 4 | 4 | 1 | Possible |
| 10013911 | Dysgeusia | 10 | 11 | 1 | Unlikely |
| 10012735 | Diarrhoea | 11 | 11 | 1 | Unlikely |
| 10028411 | Myalgia | 15 | 15 | 2 | Unlikely |
| 10061428 | Decreased appetite | 19 | 20 | 2 | Unlikely |
| 10028813 | Nausea | 19 | 19 | 1 | Unlikely |
| 10068319 | Oropharyngeal pain | 20 | 20 | 1 | Unlikely |
| 10016256 | Fatigue | 20 | 20 | 1 | Unlikely |
| 10012378 | Depression | 20 | 20 | 1 | Unlikely |
| 10046571 | Urinary tract infection | 20 | 23 | 1 | Unlikely |
| 10047700 | Vomiting | 23 | 24 | 3 | Unlikely |
| 10012735 | Diarrhoea | 24 | 24 | 3 | Unlikely |
| 10019211 | Headache | 24 | 24 | 2 | Unlikely |
| 10022004 | Influenza like illness | 24 | 24 | 1 | Unlikely |
| 10010741 | Conjunctivitis | 24 | 24 | 1 | Unlikely |
| 10016256 | Fatigue | 24 | 24 | 2 | Unlikely |
| 10025482 | Malaise | 25 | 25 | 1 | Unlikely |

**D: Group 2 Vaccine 2**

| **PT code** | **Meddra PT** | **Day of onset (relative to vaccination)** | **Last day reported (relative to vaccination)** | **Max Severity** | **Causality** |
| --- | --- | --- | --- | --- | --- |
| 10017553 | Furuncle | 0 | 27 | 1 | Possible |
| 10012735 | Diarrhoea | 0 | 1 | 1 | Possible |
| 10061428 | Decreased appetite | 1 | 4 | 1 | Possible |
| 10017553 | Furuncle | 0 | 5 | 1 | Possible |
| 10000087 | Abdominal pain upper | 5 | 5 | 1 | Possible |
| 10037844 | Rash | 6 | 25 | 1 | Possible |
| 10068319 | Oropharyngeal pain | 7 | 8 | 1 | Unlikely |
| 10019211 | Headache | 9 | 9 | 1 | Unlikely |
| 10067152 | Oral herpes | 11 | 15 | 1 | Unrelated |
| 10019211 | Headache | 11 | 11 | 1 | Unlikely |
| 10028810 | Nasopharyngitis | 13 | 18 | 2 | Unlikely |
| 10019211 | Headache | 13 | 14 | 1 | Unlikely |
| 10019211 | Headache | 13 | 13 | 1 | Unlikely |
| 10019211 | Headache | 20 | 20 | 1 | Unlikely |
| 10052136 | Ear congestion | 21 | 27 | 1 | Unlikely |
| 10019211 | Headache | 22 | 22 | 2 | Unlikely |
| 10028735 | Nasal congestion | 24 | 24 | 1 | Unlikely |
| 10019211 | Headache | 24 | 24 | 2 | Unlikely |
| 10003399 | Arthropod bite | 25 | 26 | 1 | Unrelated |

**E: Groups 3 & 4 Vaccine 1**

| **PT code** | **Meddra PT** | **Day of onset (relative to vaccination)** | **Last day reported (relative to vaccination)** | **Max Severity** | **Causality** |
| --- | --- | --- | --- | --- | --- |
| 10039101 | Rhinorrhoea | 0 | 0 | 2 | Possible |
| 10016326 | Feeling cold | 0 | 0 | 3 | Possible |
| 10033775 | Paraesthesia | 0 | 1 | 1 | Possible |
| 10016326 | Feeling cold | 0 | 0 | 2 | Possible |
| 10008531 | Chills | 0 | 0 | 1 | Probable |
| 10046306 | Upper respiratory tract infection | 0 | 0 | 1 | Possible |
| 10016256 | Fatigue | 0 | 0 | 1 | Probable |
|  | Chills | 0 | 0 | 2 | Probable |
| 10008531 | Chills | 0 | 0 | 2 | Probable |
| 10008531 | Chills | 0 | 0 | 2 | Probable |
| 10034568 | Peripheral coldness | 0 | 0 | 1 | Possible |
| 10008531 | Chills | 1 | 1 | 1 | Probable |
| 10020642 | Hyperhidrosis | 1 | 1 | 2 | Probable |
| 10028411 | Myalgia | 1 | 1 | 1 | Probable |
| 10003239 | Arthralgia | 1 | 1 | 1 | Possible |
| 10013573 | Dizziness | 1 | 1 | 1 | Possible |
| 10008531 | Chills | 1 | 1 | 1 | Probable |
| 10016326 | Feeling cold | 1 | 1 | 1 | Possible |
| 10015958 | Eye pain | 1 | 1 | 1 | Possible |
| 10024770 | Local swelling | 2 | 5 | 3 | Probable |
| 10050584 | Contusion | 2 | 6 | 1 | Probable |
| 10025188 | Lymphadenitis | 2 | 5 | 2 | Probable |
| 10015150 | Erythema | 2 | 4 | 2 | Probable |
| 10013935 | Dysmenorrhoea | 2 | 2 | 1 | Possible |
| 10014020 | Ear pain | 2 | 2 | 1 | Possible |
| 10037844 | Rash | 5 | 8 | 1 | Possible |
| 10013946 | Dyspepsia | 6 | 6 | 2 | Possible |
| 10039083 | Rhinitis | 7 | 8 | 1 | Unlikely |
| 10068881 | Vaccination site pruritus | 7 | 7 | 1 | Probable |
| 10019211 | Headache | 7 | 7 | 2 | Unlikely |
| 10019211 | Headache | 7 | 7 | 1 | Unlikely |
| 10040753 | Sinusitis | 8 | 15 | 2 | Unlikely |
| 10016256 | Fatigue | 8 | 9 | 1 | Possible |
| 10013946 | Dyspepsia | 9 | 9 | 2 | Unlikely |
| 10052576 | Pruritus generalised | 11 | 12 | 2 | Possible |
| 10068319 | Oropharyngeal pain | 14 | 17 | 1 | Unlikely |
| 10011224 | Cough | 14 | 26 | 2 | Unlikely |
| 10019211 | Headache | 15 | 18 | 2 | Unlikely |
| 10028411 | Myalgia | 16 | 17 | 1 | Possible |
| 10016256 | Fatigue | 16 | 18 | 2 | Possible |
| 10025482 | Malaise | 16 | 16 | 1 | Possible |
| 10037660 | Pyrexia | 16 | 16 | 1 | Possible |
| 10068319 | Oropharyngeal pain | 16 | 16 | 1 | Unlikely |
| 10037660 | Pyrexia | 16 | 19 | 2 | Possible |
| 10019211 | Headache | 16 | 17 | 2 | Unlikely |
| 10028411 | Myalgia | 16 | 19 | 1 | Possible |
| 10028813 | Nausea | 16 | 17 | 2 | Unlikely |
| 10028836 | Neck pain | 17 | 18 | 1 | Unlikely |
| 10003239 | Arthralgia | 16 | 19 | 1 | Unlikely |
| 10028735 | Nasal congestion | 18 | 19 | 1 | Unlikely |
| 10019211 | Headache | 19 | 25 | 2 | Unlikely |
| 10069489 | Vaccination site exfoliation | 19 | 19 | 1 | Possible |
| 10046306 | Upper respiratory tract infection | 19 | 22 | 1 | Unlikely |
| 10028735 | Nasal congestion | 20 | 23 | 1 | Unlikely |
| 10019211 | Headache | 20 | 22 | 2 | Unlikely |
| 10068319 | Oropharyngeal pain | 23 | 26 | 1 | Unlikely |
| 10028735 | Nasal congestion | 23 | 23 | 1 | Unlikely |
| 10011224 | Cough | 23 | 27 | 1 | Unlikely |
| 10016256 | Fatigue | 23 | 24 | 1 | Unlikely |
| 10039083 | Rhinitis | 24 | 27 | 1 | Unlikely |
| 10039083 | Rhinitis | 25 | 27 | 1 | Unlikely |
| 10019211 | Headache | 25 | 25 | 1 | Unlikely |
| 10048865 | Hypoacusis | 25 | 26 | 1 | Unlikely |
| 10019211 | Headache | 25 | 25 | 1 | Unlikely |
| 10028735 | Nasal congestion | 25 | 27 | 1 | Unlikely |
| 10011224 | Cough | 25 | 27 | 1 | Unlikely |
| 10040753 | Sinusitis | 26 | 27 | 1 | Unlikely |
| 10013573 | Dizziness | 26 | 26 | 1 | Unlikely |
| 10019211 | Headache | 27 | 27 | 1 | Unlikely |
| 10016256 | Fatigue | 27 | 27 | 1 | Unlikely |

**F: Groups 3 & 4 Vaccine 2**

| **PT code** | **Meddra PT** | **Day of onset (relative to vaccination)** | **Last day reported (relative to vaccination)** | **Max Severity** | **Causality** |
| --- | --- | --- | --- | --- | --- |
| 10013573 | Dizziness | 0 | 0 | 1 | Possible |
| 10069620 | Vaccination site swelling | 0 | 0 | 1 | Definite |
| 10008531 | Chills | 0 | 0 | 1 | Probable |
| 10013578 | Dizziness postural | 0 | 1 | 2 | Possible |
| 10021113 | Hypothermia | 0 | 1 | 2 | Possible |
| 10040753 | Sinusitis | 0 | 8 | 1 | Possible |
| 10052136 | Ear congestion | 0 | 1 | 1 | Possible |
| 10008531 | Chills | 0 | 0 | 2 | Probable |
| 10013573 | Dizziness | 0 | 0 | 1 | Possible |
| 10008531 | Chills | 0 | 0 | 2 | Probable |
| 10008531 | Chills | 0 | 0 | 2 | Probable |
| 10022437 | Insomnia | 0 | 0 | 1 | Possible |
| 10029446 | Nocturia | 0 | 3 | 1 | Possible |
| 10003549 | Asthenia | 0 | 0 | 3 | Probable |
| 10008531 | Chills | 0 | 0 | 1 | Probable |
| 10008531 | Chills | 0 | 1 | 2 | Probable |
| 10008531 | Chills | 1 | 1 | 2 | Probable |
| 10036790 | Productive cough | 1 | 1 | 1 | Possible |
| 10008531 | Chills | 1 | 1 | 1 | Probable |
| 10011224 | Cough | 1 | 1 | 1 | Possible |
| 10012735 | Diarrhoea | 1 | 1 | 2 | Possible |
| 10040984 | Sleep disorder | 1 | 1 | 3 | Probable |
| 10008531 | Chills | 1 | 1 | 3 | Probable |
| 10047700 | Vomiting | 1 | 1 | 1 | Possible |
| 10043071 | Tachycardia | 1 | 1 | 1 | Possible |
| 10014020 | Ear pain | 2 | 6 | 2 | Possible |
| 10069484 | Vaccination site bruising | 2 | 6 | 1 | Probable |
| 10061428 | Decreased appetite | 2 | 6 | 1 | Possible |
| 10059133 | Cluster headache | 4 | 4 | 2 | Possible |
| 10068319 | Oropharyngeal pain | 4 | 7 | 1 | Possible |
| 10028735 | Nasal congestion | 5 | 7 | 2 | Possible |
| 10036790 | Productive cough | 6 | 7 | 1 | Possible |
| 10069484 | Vaccination site bruising | 6 | 6 | 1 | Probable |
| 10028836 | Neck pain | 6 | 20 | 2 | Possible |
| 10019211 | Headache | 7 | 7 | 1 | Unlikely |
| 10016256 | Fatigue | 7 | 7 | 1 | Possible |
| 10003239 | Arthralgia | 7 | 21 | 2 | Unrelated |
| 10028391 | Musculoskeletal pain | 7 | 14 | 2 | Possible |
| 10047700 | Vomiting | 9 | 9 | 3 | Unlikely |
| 10012735 | Diarrhoea | 9 | 9 | 3 | Unlikely |
| 10019211 | Headache | 10 | 10 | 1 | Unlikely |
| 10018870 | Blood urine present | 10 | 12 | 2 | Unlikely |
| 10028813 | Nausea | 10 | 11 | 1 | Unlikely |
| 10059133 | Cluster headache | 11 | 12 | 2 | Unlikely |
| 10037844 | Rash | 12 | 12 | 1 | Possible |
| 10017553 | Furuncle | 12 | 122 | 1 | Unlikely |
| 10016256 | Fatigue | 12 | 12 | 2 | Possible |
| 10019211 | Headache | 13 | 13 | 1 | Unlikely |
| 10039083 | Rhinitis | 14 | 15 | 1 | Unlikely |
| 10052140 | Eye pruritus | 16 | 16 | 1 | Unlikely |
| 10052904 | Musculoskeletal stiffness | 16 | 16 | 1 | Unlikely |
| 10020568 | Hyperaesthesia | 17 | 17 | 1 | Unlikely |
| 10074170 | Candida infection | 18 | 18 | 1 | Unlikely |
| 10047700 | Vomiting | 19 | 19 | 2 | Unlikely |
| 10028813 | Nausea | 19 | 20 | 2 | Unlikely |
| 10016256 | Fatigue | 19 | 20 | 2 | Unlikely |
| 10028391 | Musculoskeletal pain | 20 | 20 | 1 | Unlikely |

**G: Group 3 Vaccine 3**

| **PT code** | **Meddra PT** | **Day of onset (relative to vaccination)** | **Last day reported (relative to vaccination)** | **Max Severity** | **Causality** |
| --- | --- | --- | --- | --- | --- |
| 10003239 | Arthralgia | 0 | 6 | 2 | Possible |
| 10003988 | Back pain | 1 | 1 | 1 | Possible |
| 10013573 | Dizziness | 1 | 1 | 2 | Possible |
| 10061458 | Feeling of body temperature change | 1 | 1 | 1 | Probable |
| 10040880 | Skin irritation | 1 | 1 | 2 | Possible |
| 10008531 | Chills | 1 | 1 | 2 | Probable |
| 10000087 | Abdominal pain upper | 1 | 1 | 2 | Possible |
| 10037844 | Rash | 2 | 2 | 1 | Possible |
| 10015150 | Erythema | 2 | 4 | 2 | Probable |
| 10059133 | Cluster headache | 3 | 3 | 2 | Possible |
| 10015150 | Erythema | 3 | 3 | 1 | Possible |
| 10059133 | Cluster headache | 9 | 12 | 3 | Unlikely |
| 10033425 | Pain in extremity | 14 | 14 | 1 | Possible |
| 10016256 | Fatigue | 14 | 15 | 1 | Possible |
| 10059133 | Cluster headache | 17 | 17 | 3 | Unlikely |
| 10016256 | Fatigue | 19 | 19 | 1 | Unlikely |
| 10019211 | Headache | 19 | 19 | 2 | Unlikely |
| 10059133 | Cluster headache | 23 | 23 | 3 | Unlikely |
| 10019211 | Headache | 24 | 24 | 1 | Unlikely |
| 10028411 | Myalgia | 25 | 27 | 1 | Unlikely |
| 10028836 | Neck pain | 27 | 27 | 2 | Unrelated |
| 10019211 | Headache | 27 | 27 | 2 | Unlikely |
| 10003988 | Back pain | 27 | 27 | 1 | Unrelated |

**H: Group 4 Vaccine 3**

| **PT code** | **Meddra PT** | **Day of onset (relative to vaccination)** | **Last day reported (relative to vaccination)** | **Max Severity** | **Causality** |
| --- | --- | --- | --- | --- | --- |
| 10043521 | Throat irritation | 0 | 0 | 1 | Possible |
| 10016326 | Feeling cold | 0 | 0 | 1 | Possible |
| 10028836 | Neck pain | 0 | 8 | 1 | Possible |
| 10016322 | Feeling abnormal | 0 | 1 | 1 | Possible |
| 10029446 | Nocturia | 1 | 3 | 1 | Possible |
| 10033557 | Palpitations | 1 | 1 | 1 | Possible |
| 10069481 | Vaccination site paraesthesia | 1 | 1 | 1 | Probable |
| 10028810 | Nasopharyngitis | 2 | 7 | 2 | Possible |
| 10047700 | Vomiting | 2 | 2 | 2 | Possible |
| 10068319 | Oropharyngeal pain | 2 | 4 | 2 | Possible |
| 10068319 | Oropharyngeal pain | 6 | 6 | 1 | Possible |
| 10013573 | Dizziness | 7 | 7 | 1 | Possible |
| 10015150 | Erythema | 7 | 7 | 1 | Possible |
| 10036790 | Productive cough | 8 | 9 | 1 | Unlikely |
| 10015090 | Epistaxis | 8 | 8 | 1 | Unlikely |
| 10016256 | Fatigue | 9 | 9 | 1 | Possible |
| 10028810 | Nasopharyngitis | 9 | 12 | 1 | Unlikely |
| 10019211 | Headache | 10 | 10 | 1 | Unlikely |
| 10068319 | Oropharyngeal pain | 9 | 12 | 1 | Unlikely |
| 10016256 | Fatigue | 11 | 11 | 1 | Possible |
| 10028735 | Nasal congestion | 9 | 12 | 1 | Unlikely |
| 10028813 | Nausea | 13 | 13 | 1 | Unlikely |
| 10028411 | Myalgia | 17 | 17 | 1 | Unlikely |
| 10028391 | Musculoskeletal pain | 19 | 19 | 1 | Unlikely |
| 10068319 | Oropharyngeal pain | 19 | 25 | 1 | Unlikely |
| 10011224 | Cough | 21 | 27 | 1 | Unlikely |
| 10074170 | Candida infection | 25 | 27 | 2 | Unlikely |
| 10016256 | Fatigue | 26 | 26 | 1 | Unlikely |

**Table S3: Unsolicited adverse events reported in the 28 day period following each vaccination with Meddra preferred term (PT) and code, day of onset relative to vaccination, last day AE reported, maximum reported severity and causality assessment outcome of relationship to vaccination. Severity grading: 1=mild; 2=moderate; 3=severe. A: Vaccination 1. Group 1 & 2 subjects received a full dose of RTS,S/AS01_B_ only. B: Vaccination 2. Group 1 & 2 subjects received a full dose of RTS,S/AS01_B_ only C: Vaccination 3. Group 1 subjects received a full dose of RTS,S/AS01_B_ only. D: Vaccination 3. Group 2 subjects received a fractional dose of RTS,S/AS01_B_ at 1/5^th^ standard dose only. E: Vaccination 1. Group 3 & 4 received a full dose of RTS,S/AS01_B_ with a concomitant dose of ChAd63 ME-TRAP. F: Vaccination 2. Group 3 & 4 received a full dose of RTS,S/AS01_B_ with a concomitant dose of MVA ME-TRAP. G: Vaccination 3. Group 3 received a full dose of RTS,S/AS01_B_ with a concomitant dose of MVA ME-TRAP. H: Vaccination 3. Group 4 received a fractional dose of RTS,S/AS01_B_ at 1/5^th^ standard dose with a concomitant dose of MVA ME-TRAP**

## Solicited AE Comparison

**A.**

|  |  | **Vaccine 1** | **Vaccine 2** | **Vaccine 3** | | |
| --- | --- | --- | --- | --- | --- | --- |
|  |  | **Group 1&2 vs Group 3&4** | **Group 1&2 vs Group 3&4** | **Group 1 vs Group 2** | **Group 3 vs Group 4** | **Group 1&2 vs Group 3&4** |
|  |  | ***p*** | ***p*** | ***p*** | ***p*** | ***p*** |
| **Pain** | **Any** | 0.2317 | 0.731 | 0.3034 | 0.582 | 0.7164 |
|  | **Severe** | >0.9999 | >0.9999 | >0.9999 | >0.9999 | >0.9999 |
| **Warmth** | **Any** | 0.1971 | 0.2049 | 0.3698 | 0.3498 | 0.2049 |
|  | **Severe** | >0.9999 | >0.9999 | >0.9999 | >0.9999 | >0.9999 |
| **Swelling** | **Any** | >0.9999 | 0.0083 | >0.9999 | >0.9999 | 0.1818 |
|  | **Severe** | >0.9999 | 0.4872 | >0.9999 | >0.9999 | 0.4872 |
| **Redness** | **Any** | >0.9999 | 0.3332 | 0.2105 | >0.9999 | 0.0057 |
|  | **Severe** | >0.9999 | >0.9999 | >0.9999 | >0.9999 | >0.9999 |
| **Itch** | **Any** | 0.606 | 0.1274 | 0.0867 | 0.1409 | 0.4506 |
|  | **Severe** | >0.9999 | 0.1274 | >0.9999 | >0.9999 | >0.9999 |
| **Fever** | **Any** | 0.0005 | 0.2553 | 0.2105 | >0.9999 | >0.9999 |
|  | **Severe** | >0.9999 | 0.2308 | >0.9999 | >0.9999 | >0.9999 |
| **Feverishness** | **Any** | 0.0578 | 0.1818 | 0.0108 | 0.6499 | 0.0536 |
|  | **Severe** | 0.2317 | 0.605 | 0.4737 | >0.9999 | 0.4872 |
| **Myalgia** | **Any** | 0.5303 | 0.7164 | 0.3698 | 0.1409 | 0.3332 |
|  | **Severe** | >0.9999 | 0.605 | 0.3698 | >0.9999 | >0.9999 |
| **Arthralgia** | **Any** | 0.0005 | 0.5231 | 0.3498 | >0.9999 | 0.5145 |
|  | **Severe** | >0.9999 | >0.9999 | >0.9999 | >0.9999 | >0.9999 |
| **Headache** | **Any** | 0.2146 | 0.1552 | 0.6499 | 0.6563 | 0.5231 |
|  | **Severe** | 0.4878 | >0.9999 | >0.9999 | >0.9999 | >0.9999 |
| **Fatigue** | **Any** | 0.3264 | 0.4075 | 0.6499 | 0.582 | 0.176 |
|  | **Severe** | >0.9999 | >0.9999 | >0.9999 | >0.9999 | >0.9999 |
| **Nausea** | **Any** | 0.2379 | 0.3332 | >0.9999 | 0.582 | >0.9999 |
|  | **Severe** | >0.9999 | >0.9999 | >0.9999 | >0.9999 | >0.9999 |
| **Malaise** | **Any** | 0.0126 | 0.6948 | 0.1789 | 0.3698 | 0.7524 |
|  | **Severe** | >0.9999 | >0.9999 | 0.4737 | >0.9999 | 0.4872 |
| **Any Solicited AE** | **Any** | 0.4878 | >0.9999 | 0.4737 | >0.9999 | 0.2308 |
|  | **Severe** | 0.0931 | 0.3008 | 0.4737 | >0.9999 | >0.9999 |

**B.**

|  | **1^st^ Vaccination** | | **2^nd^ Vaccination** | | **3^rd^ Vaccination** | |
| --- | --- | --- | --- | --- | --- | --- |
|  | **n (%)** | ***p*** | **n (%)** | ***p*** | **n (%)** | ***p*** |
| **Group 1 & 2** | 0 | 1 | 2 (10) | 0.6614 | 1 (5.3) | 1 |
| **Group 3 & 4** | 1 (4.8) |  | 4 (20) |  | 1 (5.0) |  |
| **Group 1** |  |  |  |  | 1 (11.1) | 0.4737 |
| **Group 2** |  |  |  |  | 0 |  |
| **Group 3** |  |  |  |  | 1 (10) | 1 |
| **Group 4** |  |  |  |  | 0 |  |

**Table S4: A: Comparison of rates of solicited adverse events between Groups as measured for 7 days following each vaccination. Rates of occurrence were compared by Fisher’s Exact test and the respective p-values are shown. Comparisons with a p-value <0.05 are highlighted in green. B. Number of subjects with at least one report of a grade 3 unsolicited AE in the 28 day period post each vaccination (Days 0-28). 2-tailed p value obtained using Fisher’s exact test. Vaccination 1: Group 1 & 2 subjects received a full dose of RTS,S/AS01_B_ only; Group 3 & 4 received a full dose of RTS,S/AS01_B_ with a concomitant dose of ChAd63 ME-TRAP. Vaccination 2: Group 1 & 2 subjects received a full dose of RTS,S/AS01_B_ only; Group 3 & 4 received a full dose of RTS,S/AS01_B_ with a concomitant dose of MVA ME-TRAP. Vaccination 3: Group 1 subjects received a full dose of RTS,S/AS01_B_ only; Group 2 subjects received a fractional dose of RTS,S/AS01_B_ at 1/5^th^ standard dose only; Group 3 received a full dose of RTS,S/AS01_B_ with a concomitant dose of MVA ME-TRAP; Group 4 received a fractional dose of RTS,S/AS01_B_ at 1/5^th^ standard dose with a concomitant dose of MVA ME-TRAP**

## Laboratory AEs

|  |  |  | **Timepoint at which first identified** | | | |
| --- | --- | --- | --- | --- | --- | --- |
|  |  |  | **Post-Vaccine 1** | **Post-Vaccine 2** | **Post-Vaccine 3** | **Post-CHMI** |
| **Group** | **AE** | **Max severity** | **n (%)** | **n (%)** | **n (%)** | **n (%)** |
| **1** | **Anaemia** | **Mild** |  |  |  | 1 (12.5) |
|  | **Eosinophilia** | **Mild** |  | 1 (10) |  |  |
|  | **Hypokalaemia** | **Mild** | 1 (10) |  |  |  |
|  | **Lymphopenia** | **Mild** |  |  | 1 (11.1) |  |
|  |  | **Moderate** |  |  |  | 1 (12.5) |
|  |  | **Severe** |  |  |  | 1 (12.5) |
|  | **Raised ALT** | **Mild** | 1 (10) | 1 (10) |  |  |
|  | **Uraemia** | **Mild** | 1 (10) |  |  |  |
| **2** | **Anaemia** | **Mild** |  |  |  | 1 (11.1) |
|  | **Hypokalaemia** | **Mild** |  |  |  | 1 (11.1) |
|  |  | **Moderate** | 1 (10) |  | 1 (10) | 1 (11.1) |
|  | **Leukocytosis** | **Mild** |  |  |  | 1 (11.1) |
|  | **Lymphopenia** | **Moderate** |  |  |  | 1 (11.1) |
|  | **Raised ALT** | **Mild** |  |  |  | 1 (11.1) |
|  |  | **Moderate** | 1 (10) |  |  |  |
|  | **Thrombocytopaenia** | **Moderate** | 1 (10) |  |  |  |
| **3** | **Anaemia** | **Mild** |  |  |  | 2 (20) |
|  | **Eosinophilia** | **Moderate** |  | 1 (10) |  |  |
|  | **Hypokalaemia** | **Mild** |  |  |  | 1 (10) |
|  |  | **Moderate** |  |  |  | 1 (10) |
|  | **Lymphopaenia** | **Mild** |  |  |  | 1 (10) |
|  |  | **Severe** |  |  |  | 3 (30) |
|  | **Neutropaenia** | **Mild** |  |  |  | 2 (20) |
|  | **Raised ALT** | **Moderate** |  |  | 1 (10)* |  |
|  |  | **Severe** |  |  |  | 1 (10) |
| **4** | **Anaemia** | **Mild** | 1 (9.1) |  |  |  |
|  |  | **Moderate** |  | 1 (10) |  |  |
|  | **Hypokalaemia** | **Mild** |  | 1 (10) |  |  |
|  | **Leukopenia** | **Mild** | 1 (9.1) |  |  |  |
|  | **Lymphopaenia** | **Moderate** | 1 (9.1)* | 1 (10) | 1 (10)* |  |
|  | **Neutropaenia** | **Mild** | 3 (27.2) |  | 1 (10) | 1 (11.1) |
|  | **Raised ALT** | **Mild** | 1 (9.1) |  |  |  |
| **5** | **Hyperbilirubineamia** | **Moderate** |  |  |  | 1 (25) |
|  | **Hypokalaemia** | **Mild** |  |  |  | 1 (25) |
|  | **Lymphopaenia** | **Severe** |  |  |  | 1 (25) |

**Table S5: Table of all laboratory adverse events identified throughout this trial, and the timepoint at which the abnormality was first identified. The table shows all events in which a laboratory value has deviated by at least 1 degree of severity from the baseline (screening) value as defined in the laboratory adverse event grading tables that can be found in the appendix. *denotes events at which the laboratory value lies within the moderate adverse event range, but was in the mild adverse event range at screening. Vaccination 1: Group 1 & 2 subjects received a full dose of RTS,S/AS01_B_ only; Group 3 & 4 received a full dose of RTS,S/AS01_B_ with a concomitant dose of ChAd63 ME-TRAP. Vaccination 2: Group 1 & 2 subjects received a full dose of RTS,S/AS01_B_ only; Group 3 & 4 received a full dose of RTS,S/AS01_B_ with a concomitant dose of MVA ME-TRAP. Vaccination 3: Group 1 subjects received a full dose of RTS,S/AS01_B_ only; Group 2 subjects received a fractional dose of RTS,S/AS01_B_ at 1/5^th^ standard dose only; Group 3 received a full dose of RTS,S/AS01_B_ with a concomitant dose of MVA ME-TRAP; Group 4 received a fractional dose of RTS,S/AS01_B_ at 1/5^th^ standard dose with a concomitant dose of MVA ME-TRAP. CHMI = Controlled Humann Malaria Infection; ALT = Alanine aminotransferase**

|  | **Malaria diagnosis** | | **Time to 20p/ml** | | **Time to 500p/ml** | |
| --- | --- | --- | --- | --- | --- | --- |
|  | **Logrank *p*** | **HR [CI]** | **Logrank *p*** | **HR [CI]** | **Logrank *p*** | **HR [CI]** |
| **Gp1 vs controls** | **0.0003** | **0.1136**  **[0.0145-0.8915** | **0.0024** | **0.1351**  **[0.0191-0.9547]** | **0.0023** | **0.1429**  **[0.0209-0.979]** |
| **Gp2 vs controls** | **0.0002** | **0.0643**  **[0.0073-0.5648]** | **0.0001** | **0.0555**  **[0.0057-0.5389]** | **0.0002** | **0.0643**  **[0.0073-0.5648]** |
| **Gp3 vs controls** | **< 0.0001** | 0.1344  [0.0158-1.147] | **< 0.0001** | 0.1170  [0.0122-1.125] | **0.0052** | 0.2200  [0.0363-1.334] |
| **Gp4 vs controls** | **0.0002** | 0.1466  [0.0184-1.168] | **0.0004** | 0.1441  [0.0179-1.163] | **0.0020** | 0.2015  [0.0315-1.288] |
| **Gp1 vs Gp2** | 0.4369 | 2.459  [0.2527-23.93] | 0.4369 | 2.459  [0.2527-23.93] | 0.4334 | 2.474  [0.254-24.09] |
| **Gp1 vs Gp3** | 0.4369 | 0.5079  [0.1025-2.516] | 0.5916 | 0.6511  [0.1296-3.271] | 0.4902 | 0.5759  [0.1158-2.865] |
| **Gp3 vs Gp4** | 0.8487 | 0.8830  [0.2202-3.54] | 0.8598 | 0.8917  [0.2225-3.574] | 0.4902 | 0.9943  [0.2487-3.976] |
| **Gp2 vs Gp4** | 0.0992 | 0.1969  [0.0337-1.151] | 0.1215 | 0.2200  [0.0380-1.274] | 0.1215 | 0.2200  [0.0380-1.274] |
| **Gp1&2 vs controls** | **< 0.0001** | **0.0738**  **[0.0055-0.9877]** | **< 0.0001** | **0.0832**  **[0.0070-0.9846]** | **< 0.0001** | **0.0884**  **[0.0079-0.9863]** |
| **Gp3&4 vs controls** | **< 0.0001** | 0.1001  [0.0070-1.425] | **< 0.0001** | 0.09546  [0.0063-1.44] | **0.0005** | 0.1890  [0.0252-1.417] |
| **Gp1&2 vs Gp3&4** | 0.0744 | 0.3298  [0.1009-1.078] | 0.1334 | 0.3986  [0.1222-1.3] | 0.1191 | 0.3773  [0.1157-1.23] |

**Table S6: Statistical analysis of the Primary and Secondary efficacy endpoints. Logrank survival analysis has been performed on each of the 3 secondary efficacy endpoints p values and hazard ratios with 95% confidence intervals on the per protocol cohorts for each of the 3 secondary efficacy endpoints.**

| **Peptide number** | **Amino acid sequence** |
| --- | --- |
| 1 | MMAP DPNANPNANPN |
| 2 | NANP NANPNANPNAN |
| 3 | DPNA NPNANPNKNNQ |
| 4 | NPNA NPNKNNQGNGQ |
| 5 | NPNK NNQGNGQGHNM |
| 6 | NNQG NGQGHNMPNDP |
| 7 | NGQG HNMPNDPNRNV |
| 8 | HNMP NDPNRNVDENA |
| 9 | NDPN RNVDENANANS |
| 10 | RNVD ENANANSAVKN |
| 11 | ENAN ANSAVKNNNNE |
| 12 | ANSA VKNNNNEEPSD |
| 13 | VKNN NNEEPSDKHIK |
| 14 | NNEE PSDKHIKEYLN |
| 15 | PSDK HIKEYLNKIQN |
| 16 | HIKE YLNKIQNSLST |
| 17 | YLNK IQNSLSTEWSP |
| 18 | IQNS LSTEWSPCSVT |
| 19 | LSTE WSPCSVTCGNG |
| 20 | WSPC SVTCGNGIQVR |
| 21 | SVTC GNGIQVRIKPG |
| 22 | GNGI QVRIKPGSANK |
| 23 | QVRI KPGSANKPKDE |
| 24 | KPGS ANKPKDELDYA |
| 25 | ANKP KDELDYANDIE |
| 26 | KDEL DYANDIEKKIC |
| 27 | DYAN DIEKKICKMEK |
| 28 | DIEK KICKMEKCSSV |
| 29 | KICK MEKCSSVFNVV |
| 30 | MEKC SSVFNVVNSSI |
| 31 | KCSS VFNVVNSSIGL |

Table S7: CSP peptides sequences. Peptide sequences were based on P. falciparum clone 3D7 (GenBank no. X15363). Series of 15 amino acid sequences overlapping by 11 amino acids.

| Pool | CSP aa | No. peptides | Protein region |
| --- | --- | --- | --- |
| Cp1 | 1-39 | 7 | NANP and conserved region |
| Cp2 | 29-71 | 8 | TH2R region |
| Cp3 | 61-107 | 9 | TH3R/CS.T3T region |

Table S8: CSP peptide pools.

| Peptide Name | Peptide sequence T9/96 | Peptide sequence 3D7  (if different from T9/96). | T9/96 Peptide Pool | 3D7 Peptide Pool |
| --- | --- | --- | --- | --- |
| TRAP-1 | MNHLGNVKYLVIVFLIFFDL |  | TT1-10 | TD1-10 |
| TRAP-2 | VIVFLIFFDLFLVNGRDVQN |  | TT1-10 | TD1-10 |
| TRAP-3 | FLVNGRDVQNNIVDEIKYSE | FLVNGRDVQNNIVDEIKYRE | TT1-10 | TD1-10 |
| TRAP-4 | NIVDEIKYSEEVCNDQVDLY | NIVDEIKYREEVCNDEVDLY | TT1-10 | TD1-10 |
| TRAP-5 | EVCNDQVDLYLLMCSGSIR | EVCNDEVDLYLLMCSGSIR | TT1-10 | TD1-10 |
| TRAP-6 | LLMCSGSIRRHNWVNHAVP |  | TT1-10 | TD1-10 |
| TRAP-7 | RHNWVNHAVPLAMKLIQQLN |  | TT1-10 | TD1-10 |
| TRAP-8 | LAMKLIQQLNLNDNAIHLYV | LAMKLIQQLNLNDNAIHLYA | TT1-10 | TD1-10 |
| TRAP-9 | LNDNAIHLYVNVFSNNAKEI | LNDNAIHLYASVFSNNAREI | TT1-10 | TD1-10 |
| TRAP-10 | LNDNAIHLYVNVFSNNAKEI | SVFSNNAREIIRLHSDASKN | TT1-10 | TD1-10 |
| TRAP-11 | IRLHSDASKNKEKALIIIRS | IRLHSDASKNKEKALIIIKS | TT11-20 | TD11-20 |
| TRAP-12 | KEKALIIIRSLLSTNLPYGR | KEKALIIIKSLLSTNLPYGK | TT11-20 | TD11-20 |
| TRAP-13 | LLSTNLPYGRTNLTDALLQV | LLSTNLPYGKTNLTDALLQV | TT11-20 | TD11-20 |
| TRAP-14 | TNLTDALLQVRKHLNDRINR |  | TT11-20 | TD11-20 |
| TRAP-15 | RKHLNDRINRENANQLVVIL |  | TT11-20 | TD11-20 |
| TRAP-16 | ENANQLVVILTDGIPDSIQD |  | TT11-20 | TD11-20 |
| TRAP-17 | TDGIPDSIQDSLKESRKLSD |  | TT11-20 | TD11-20 |
| TRAP-18 | SLKESRKLSDRGVKIAVFGI |  | TT11-20 | TD11-20 |
| TRAP-19 | RGVKIAVFGIGQGINVAFNR |  | TT11-20 | TD11-20 |
| TRAP-20 | GQGINVAFNRFLVGCHPSDG |  | TT11-20 | TD11-20 |
| TRAP-21 | FLVGCHPSDGKCNLYADSAW |  | TT21-30 | TD21-30 |
| TRAP-22 | KCNLYADSAWENVKNVIGPF |  | TT21-30 | TD21-30 |
| TRAP-23 | ENVKNVIGPFMKAVCVEVEK |  | TT21-30 | TD21-30 |
| TRAP-24 | MKAVCVEVEKTASCGVWDEW |  | TT21-30 | TD21-30 |
| TRAP-25 | TASCGVWDEWSPCSVTCGKG |  | TT21-30 | TD21-30 |
| TRAP-26 | SPCSVTCGKGTRSRKREILH |  | TT21-30 | TD21-30 |
| TRAP-27 | TRSRKREILHEGCTSEIQEQ | TRSRKREILHEGCTSELQEQ | TT21-30 | TD21-30 |
| TRAP-28 | EGCTSEIQEQCEEERCPPKW | EGCTSELQEQCEEERCLPKR | TT21-30 | TD21-30 |
| TRAP-29 | CEEERCPPKWEPLDVPDEPE | CEEERCLPKREPLDVPDEPE | TT21-30 | TD21-30 |
| TRAP-30 | EPLDVPDEPEDDQPRPRGDN |  | TT21-30 | TD21-30 |
| TRAP-31 | DDQPRPRGDNSSVQKPEENI | DDQPRPRGDNFAVEKPNENI | TT31-40 | TD31-40 |
| TRAP-32 | SSVQKPEENIIDNNPQEPSP | FAVEKPNENIIDNNPQEPSP | TT31-40 | TD31-40 |
| TRAP-33 | IDNNPQEPSPNPEEGKDENP | IDNNPQEPSPNPEEGKGENP | TT31-40 | TD31-40 |
| TRAP-34 | NPEEGKDENPNGFDLDENPE | NPEEGKGENPNGFDLDENPE | TT31-40 | TD31-40 |
| TRAP-35 | NGFDLDENPENPPNPDIPEQ | NGFDLDENPENPPNPPNPPN | TT31-40 | TD31-40 |
| TRAP-36 | NPPNPDIPEQKPNIPEDSEK | NPPNPPNPPNPPNPPNPPNP | TT31-40 | TD31-40 |
| TRAP-37 | NONE | PPNPPNPPNPDIPEQKPNIP | TT31-40 | TD31-40 |
| TRAP-38 | DIPEQKPNIPEDSEKEVPSD | DIPEQKPNIPEDSEKEVPSD | TT31-40 | TD31-40 |
| TRAP-39 | EDSEKEVPSDVPKNPEDDRE |  | TT31-40 | TD31-40 |
| TRAP-40 | VPKNPEDDREENFDIPKKPE |  | TT31-40 | TD31-40 |
| TRAP-41 | ENFDIPKKPENKHDNQNNLP |  | TT41-50 | TD41-50 |
| TRAP-42 | NKHDNQNNLPNDKSDRNIPY | NKHDNQNNLPNDKSDRYIPY | TT41-50 | TD41-50 |
| TRAP-43 | NDKSDRNIPYSPLPPKVLDN | NDKSDRYIPYSPLAPKVLDN | TT41-50 | TD41-50 |
| TRAP-44 | SPLPPKVLDNERKQSDPQSQ | SPLAPKVLDNERKQSDPQSQ | TT41-50 | TD41-50 |
| TRAP-45 | ERKQSDPQSQDNNGNRHVPN |  | TT41-50 | TD41-50 |
| TRAP-46 | DNNGNRHVPNSEDRETRPHG |  | TT41-50 | TD41-50 |
| TRAP-47 | SEDRETRPHGRNNENRSYNR |  | TT41-50 | TD41-50 |
| TRAP-48 | RNNENRSYNRKYNDTPKHPE |  | TT41-50 | TD41-50 |
| TRAP-49 | KYNDTPKHPEREEHEKPDNN |  | TT41-50 | TD41-50 |
| TRAP-50 | REEHEKPDNNKKKGESDNKY |  | TT41-50 | TD41-50 |
| TRAP-51 | KKKGESDNKYKIAGGIAGGL |  | TT51-57 | TT51-57 |
| TRAP-52 | KIAGGIAGGLALLACAGLAY |  | TT51-57 | TT51-57 |
| TRAP-53 | ALLACAGLAYKFVVPGAATP |  | TT51-57 | TT51-57 |
| TRAP-54 | KFVVPGAATPYAGEPAPFDE |  | TT51-57 | TT51-57 |
| TRAP-55 | YAGEPAPFDETLGEEDKDLD |  | TT51-57 | TT51-57 |
| TRAP-56 | TLGEEDKDLDEPEQFRLPEE |  | TT51-57 | TT51-57 |
| TRAP-57 | EPEQFRLPEENEWN |  | TT51-57 | TT51-57 |

## **Table S9: TRAP peptide sequences and pools.**

| Antibody | Fluorochrome  /Dye | Clone | Supplier | Product code | Final Dilution |
| --- | --- | --- | --- | --- | --- |
|  | LIVE/DEAD  (Amine reactive dye) | AQUA | Life Technologies | L34955 | 1:400 |
| CD3 | Alexa Fluor 700 | OKT3 | eBioscience | 56-0037-42 | 1:100 |
| CD4 | Allophycocyanin (APC) | RPA-T4 | eBioscience | 17-0049-73 | 1:50 |
| CD8 | APC-eFluor780 | RPA-T8 | eBioscience | 47-0088-42 | 1:25 |
| CD14 | eFluor 450 | 61D3 | eBioscience | 48-0149 | 1:100 |
| CD19 | eFluor 450 | HIB19 | eBioscience | 48-0199 | 1:100 |
| CD107a | PE-Cy5 | eBioH4A3 | eBioscience | 15-1079-42 | 1:100 |
| IFNγ | FITC | 4S.B3 | eBioscience | 11-7319-82 | 1:200 |
| IL-2 | PE | MQ1-17H12 | eBioscience | 12-7029-82 | 1:100 |
| TNFα | PE-Cy7 | MAb11 | eBioscience | 25-7349-82 | 1:1000 |

Table S10: Antibodies used for flow cytometry. CD107a was added at the start of the 18-hour stimulation, LIVE/DEAD was surface-stained. All other markers were stained after permeabilisation.

# Supplementary Figures and Results

## Supplementary Figure SF1

**Supplementary Figure SF1:** Flow diagram of study design and volunteer recruitment. Thirteen subjects were excluded according to inclusion/exclusion criteria. Twelve subjects withdrew consent after screening, but before enrolment. Four subjects were deemed eligible as control subjects but only after Group 5 enrolment was complete. They were kept as backup subjects in case of last minute withdrawals from Group 5, but never underwent CHMI. Prior to CHMI, 2 subjects withdrew from group 1, 1 subject withdrew from Group 2, and 2 subjects withdrew from Group 4. There were no withdrawals due to safety concerns and no pre-defined study stopping or holding rules were activated.A single subject had received vaccination with a non-malaria viral vectored vaccine, and was therefore randomized to Group 1 or 2 only. The remaining 40 vaccine recipients were randomized across the 4 vaccine study groups by the study statistician. RTS,S, full standard dose of RTS,S/AS01B (50μg); RTS,S* fractional dose of RTS,S/AS01B (10μg); ChAd63, Chimpanzee adenovirus serotype 63 expressing multiple-epitope thrombospondin-related adhesion protein (ME-TRAP); MVA, Modified vaccinia virus Ankara expressing ME-TRAP; CHMI, Controlled human malaria infection.

# Supplementary T cell immunogenicity

CSP-specific T cell frequencies were low, with highest measured responses at day 42, 2 weeks after the second dose of RTS,S in all groups. There were no significant differences between any individual groups (p=0.75, Kruskal-Wallis test) nor between groups that received the fractional third dose of RTS,S compared with the full dose (p=0.5, 2-tailed Mann-Whitney test comparing groups 1 and 3 with groups 2 and 4, SF1A). After day 42, responses across all four groups decreased significantly from a median of 89 spot-forming cells (SFC) per million peripheral blood mononuclear cells (PBMC), interquartile range (IQR) 31-156 SFC at day 42 to 28 SFC (IQR 12-117 SFC) at the day before challenge (C-1) (p=0.009, 2-tailed Wilcoxon test).

## Supplementary Figure 2.

**Supplementary Figure SF2. Antigen-specific T cell responses to vaccination enumerated by IFNγ ELISPOT.** A, Median response to summed circumsporozoite protein (CSP) peptide pools. B, Median response to summed peptide pools for multi-epitope string fused to thrombospondin-related adhesion protein (ME-TRAP). C, ME-TRAP-specific T cell responses one week after one or two doses of MVA co-administered with RTS,S/AS01B or viral vectors alone, * P=0.04 one-way ANOVA. Data shown are combined with previous studies of the same vaccines (see section 3.8 Supplementary information) [1, 2]. R, full dose RTS,S/AS01B; A, ChAd63 ME-TRAP; M, MVA ME-TRAP; r fractional dose RTS,S/AS01B; CHMI, controlled human malaria infection; SFCs, spot-forming cells per million PBMC.

## Supplementary Figure 3.

**Supplementary Figure SF3. T cell responses characterised by flow cytometry with intracellular cytokine staining.** A, ICS responses to CSP peptides spanning the length of the vaccine antigen. B, ICS responses to TRAP peptides from either the T9/96 or 3D7 strain of *Plasmodium falciparum*. Groups 3 and 4 are combined as both received identical doses of viral vectors encoding ME TRAP. C-1, one day prior to controlled human malaria infection; LLD, lower limit of detection.

## Supplementary Figure 4

**SF4. Phenotypic analysis of TRAP-specific CD8^+^ T cell responses.**

CD8^+^ T cell responses to TRAP were dominated by cells expressing IFNg either alone or in combination with other cytokines. Group 3 tended to have more triple-positive cells than group 4. Responses did not change substantially between post-Ad and post-first MVA, but the 2nd dose of MVA substantially reduced the proportion of monofunctional cells expressing IFNg (D63 and C-1).

Supplementary ELISA results -

Antibody responses to TRAP were measured in groups 3 and 4, Figure 4C and D. Again, data for these groups were combined as there were no significant differences between them. Titres were compared with those from a previous trial where RTS,S/AS01B and viral vectors were administered two weeks apart from each other (VAC55 Figure 4C)[2]. TRAP IgG titres peaked after the initial MVA and were not re-boosted by the second MVA 4 weeks later. TRAP-specific IgG titres at peak post ChAd63 vaccination (D28) were comparable to those in the staggered regimen (Figure 4D). Peak titres after the first MVA vaccination were comparable to those after a single MVA boost at 8 weeks in the staggered regimen (V55, C-1). However, peak titres after the second MVA (V59 C-1) were significantly lower than those at the same time point in the staggered regimen (P=0.03). There were no significant differences in the post-challenge titres between the two regimens.

## Supplementary Figure 5

**Supplementary Figure SF5. Anti-vector antibody responses.** A, Relationship between anti-MVA antibodies at peak post MVA (C-1) and TRAP-specific T cell responses post MVA at C-1 in VAC55 G1, spearman’s r = 0.17, P value = 0.5. B, Relationship between anti-MVA antibodies and TRAP-specific T cell responses at peaks post-1st MVA (D42 and D35 respectively), in VAC59 G3/4, spearman’s r = 0.28, P value = 0.3. The dotted line represents the positive threshold, as calculated from the mean +3 SD of the day 0 responses for all participants.

Supplementary Figure 6

**Supplementary Figure SF6. Flow cytometry gating strategy.** Singlets were identified using forward scatter plots. Dead cells were excluded by aqua fluorescent amine-reactive dye staining. Monocytes and B cells were excluded by CD14 or CD19 expression and T cells identified by CD3 expression. T cells were then subdivided by gating on CD4+ and CD8+ populations. Cytokine expression was quantified by plotting pairs of cytokines against each other and gating positive populations. This is a representative sample from a sample stimulated overnight (18 hours) with a single pool of overlapping TRAP peptides.

# Supplementary Methods

## Study Sites

Volunteers were recruited and underwent screening visits, vaccination and clinic visits post vaccination at their local trial site; either the Centre for Clinical Vaccinology and Tropical Medicine (CCVTM), Oxford, National Institutes for Health Research (NIHR) Wellcome Trust Clinical Research Facility (WTCRF), Southampton, Surrey Clinical Research Centre, Guildford or the Hammersmith Hospital NIHR Wellcome Trust Imperial CRF London. All volunteers then travellled to Imperial College, London to undergo CHMI by mosquito bite. From days 1-5 post mosquito bite, volunteers returned to their own homes in either Oxford, Southampton, Guildford or London. From days 6.5 after CHMI until completion of a curative course of anti-malarial therapy, all volunteers resided in Oxford and attended follow-up visits at the CCVTM, Oxford. Southampton, Guildford and London volunteers were provided with accommodation in Oxford for this period. Clinic visits at days 35 and 90 post challenge took place at volunteers’ local trial site; either CCVTM, Oxford, NIHR WTCRF, Southampton, the Surrey Clinical Research Centre, Guildford or the Hammersmith Hospital NIHR Wellcome Trust Imperial CRF London.

## Objectives

### PRIMARY OBJECTIVE

To assess the efficacy (occurrence of P. falciparum parasitemia, assessed by blood slide) of a combination immunization regimen of ChAd63 ME-TRAP and MVA ME-TRAP delivered concomitantly with RTS,S/AS01B, at either full standard dose or with a fractional 3rd dose (1/5 standard dose), and of RTS,S/AS01B alone at either full standard dose or with a fractional 3rd dose, against malaria sporozoite challenge, in healthy malaria-naïve volunteers.

To assess the safety of a combination immunization regimen of ChAd63 ME-TRAP and MVA ME-TRAP delivered concomitantly with RTS,S/AS01B, at either full standard dose or with a fractional 3rd dose (1/5 standard dose), and of RTS,S/AS01B alone at either full standard dose or with a fractional 3rd dose, in healthy malaria-naïve volunteers.

### SECONDARY OBJECTIVES

To assess immunogenicity generated in malaria naïve individuals of a combination immunization regimen of ChAd63 ME-TRAP and MVA ME-TRAP delivered concomitantly with RTS,S/AS01B, at either full standard dose or with a fractional 3rd dose (1/5 standard dose), and of RTS,S/AS01B alone at either full standard dose or with a fractional 3rd dose.

To assess the efficacy (measured as time to P. falciparum parasitemia assessed by blood slide, by PCR, and parasite density dynamics assessed by PCR) of a combination immunization regimen of ChAd63 ME-TRAP and MVA ME-TRAP delivered concomitantly with RTS,S/AS01B, at either full standard dose or with a fractional 3rd dose (1/5 standard dose), and of RTS,S/AS01B alone at either full standard dose or with a fractional 3rd dose, against malaria sporozoite challenge, in healthy malaria-naïve volunteers.

## Sample Size

This was an open label, partially-randomised study with a factorial design. The target number of volunteers undergoing vaccination followed by malaria challenge was 12 for each vaccination regimen. The number of unvaccinated control volunteers was 4. Group 1, 2, 3 & 4 sizes reflect practical limitations on volunteer recruitment, ethical considerations limiting the number of volunteers that should receive a vaccine regimen without prior evidence of efficacy, and the desire to describe the efficacy of the Group 3 and 4 regimens in parallel with the efficacy of the group 1 and 2 RTS,S/AS01 only immunisation regimes.

## Inclusion/Exclusion Criteria

### Inclusion Criteria

The volunteer must satisfy all the following criteria to be eligible for the study:

- Healthy adults aged 18 to 45 years.
- Able and willing (in the Investigator’s opinion) to comply with all study requirements.
- Willing to allow the investigators to discuss the volunteer’s medical history with their General Practitioner.
- Women only: Must practice continuous effective contraception* for the duration of the study.
- Agreement to refrain from blood donation during the course of the study and for at least 3 years after the end of their involvement in the study.
- Written informed consent to participate in the trial.
- Reachable (24/7) by mobile phone during the period between CHMI and completion of antimalarial treatment.
- Willingness to take a curative anti-malaria regimen following CHMI.
- For volunteers not living in Oxford: agreement to stay in a hotel room close to the trial centre during a part of the study (from at least day 6.5 post mosquito bite until anti-malarial treatment is completed).
- Answer all questions on the informed consent quiz correctly.

* Acceptable forms of contraception include:

- Established use of oral, injected or implanted hormonal contraceptives
- Intrauterine Device or Intrauterine System
- Barrier methods (condoms or diaphragm ***with*** additional spermicide)
- Male sterilisation (with appropriate post-vasectomy documentation of absence of sperm in the ejaculate)
- True abstinence, when this is in line with the preferred and usual lifestyle of the subject. Periodic abstinence (e.g., calendar, ovulation, symptothermal, post-ovulation methods) and withdrawal are not acceptable methods of contraception.

### Exclusion Criteria

The volunteer may not enter the study if any of the following apply:

- History of clinical malaria (any species).
- Travel to a malaria endemic region during the study period or within the preceding six months with significant risk of malaria exposure.
- Use of systemic antibiotics with known antimalarial activity within 30 days of CHMI (e.g. trimethoprim-sulfamethoxazole, doxycycline, tetracycline, clindamycin, erythromycin, fluoroquinolones and azithromycin)
- Receipt of an investigational product in the 30 days preceding enrolment, or planned receipt during the study period.
- Prior receipt of an investigational malaria vaccine or any other investigational vaccine likely to impact on interpretation of the trial data. If any volunteers in Group 1-4 undergo rechallenge, this exclusion criterion does not extend to the vaccines previously received in the VAC059 trial
- Any confirmed or suspected immunosuppressive or immunodeficient state, including HIV infection; asplenia; recurrent, severe infections and chronic (more than 14 days) immunosuppressant medication within the past 6 months (inhaled and topical steroids are allowed).
- Use of immunoglobulins or blood products within 3 months prior to enrolment.
- History of allergic disease or reactions likely to be exacerbated by any component of the vaccine (e.g. egg products, Kathon) or malaria infection.
- Any history of anaphylaxis post vaccination.
- History of clinically significant contact dermatitis.
- History of sickle cell anaemia, sickle cell trait, thalassaemia or thalassaemia trait or any haematological condition that could affect susceptibility to malaria infection.
- Pregnancy, lactation or intention to become pregnant during the study.
- Use of medications known to cause prolongation of the QT interval ***and*** existing contraindication to the use of Malarone
- Use of medications known to have a potentially clinically significant interaction with Riamet ***and*** Malarone
- Any clinical condition known to prolong the QT interval
- History of cardiac arrhythmia, including clinically relevant bradycardia
- Disturbances of electrolyte balance, eg, hypokalaemia or hypomagnesaemia
- Family history of congenital QT prolongation or sudden death
- Contraindications to the use of all three proposed anti-malarial medications; Riamet, Malarone and Chloroquine.
- History of cancer (except basal cell carcinoma of the skin and cervical carcinoma in situ).
- History of serious psychiatric condition that may affect participation in the study.
- Any other serious chronic illness requiring hospital specialist supervision.
- Suspected or known current alcohol abuse as defined by an alcohol intake of greater than 42 standard UK units every week.
- Suspected or known injecting drug abuse in the 5 years preceding enrolment.
- Hepatitis B surface antigen (HBsAg) detected in serum.
- Seropositive for hepatitis C virus (antibodies to HCV) at screening (***unless*** has taken part in a prior hepatitits C vaccine study with confirmed negative HCV antibodies prior to participation in that study, and negative HCV RNA PCR at screening for this study).
- An estimated, ten year risk of fatal cardiovascular disease of ≥5%, as estimated by the Systematic Coronary Risk Evaluation (SCORE) system.[3]
- Positive family history in 1st and 2nd degree relatives < 50 years old for cardiac disease.
- Volunteers unable to be closely followed for social, geographic or psychological reasons.
- Any clinically significant abnormal finding on biochemistry or haematology blood tests, urinalysis or clinical examination. In the event of abnormal test results, confirmatory repeat tests will be requested. Procedures for identifying laboratory values meeting exclusion criteria are shown in Appendix A.

Any other significant disease, disorder, or finding which may significantly increase the risk to the volunteer because of participation in the study, affect the ability of the volunteer to participate in the study or impair interpretation of the study data.

## Vaccines

Vaccine use was authorized by the Genetically Modified Organisms Safety Committee (GMSC) of the Oxford University Hospitals NHS Trust (Reference number GM462.14.75), and the Joint Clinical Research Safety Committee of Imperial College London.

### RTS,S/AS01_B_ Vaccine

The RTS,S/AS01_B_ vaccine has been developed and manufactured by GSK Vaccines. The active substance is a recombinant antigen expressed in Saccharomyces coded RTS,S. RTS is a hybrid polypeptide consisting of a portion of the CSP antigen of the *P. falciparum* NF54 strain, fused to the amino terminal end of the hepatitis B virus surface (S) protein. S is a polypeptide corresponding to the surface antigen of hepatitis B virus (HBsAg) and is the same antigen used in GSK Vaccines’ licensed hepatitis B vaccine (Engerix-B). AS01_B_ is an Adjuvant System containing 3-*O*-desacyl-4’- monophosphoryl lipid A (MPL, 50µg, produced by GSK), *Quillaja saponaria* Molina, fraction 21 (QS-21, 50µg, Licensed by GSK from Antigenics Inc, a wholly owned subsidiary of Agenus Inc., a Delaware, USA corporation) and liposome.

### ChAd63-MVA ME-TRAP Vaccines

Both vectored vaccines in this study encoded the same insert, ME-TRAP which comprises a multiple epitope string (ME) fused to the *P. falciparum* T9/96 strain pre-erythrocytic thrombospondin-related adhesion protein (TRAP). Generation, manufacture and QC monitoring of the ChAd63 and MVA recombinant viral vectors encoding ME-TRAP has been described previously.[4]

### Vaccine administration

For concomitant administration, a circle of 1 centimetre diameter was drawn onto the skin of the deltoid muscle. Vaccines were administered intramuscularly within the circle. RTS,S/AS01_B_ vaccine was administered first within the circle, followed by the viral vector in the same circle. The interval between vaccinations was no longer than 5 minutes.

## Randomisation

Initially subjects were allowed to choose whether to be allocated to the vaccination groups or to the control group. Some subjects were ineligible for either Group 3 or 4 due to prior receipt of a viral vectored, non-malaria vaccine, and were therefore randomised to either Group 1 or 2. All remaining vaccine subjects were randomised to Groups 1-4 (target n=13 per group) to receive 3 sets of vaccinations at 4-week intervals.

## Assessment of Safety

Safety of the IMPs was assessed by analysing the frequency, incidence and nature of adverse events and serious adverse events arising during the study.

### Definitions

**Adverse Event (AE):** An AE is any untoward medical occurrence in a volunteer, including a dosing error, which may occur during or after administration of an IMP and does not necessarily have a causal relationship with the intervention. An AE can therefore be any unfavourable and unintended sign (including an abnormal laboratory finding), symptom or disease temporally associated with the study intervention, whether or not considered related to the study intervention.

**Adverse Reaction (AR):** An AR is any untoward or unintended response to an IMP. This means that a causal relationship between the IMP and an AE is at least a reasonable possibility, i.e., the relationship cannot be ruled out. All cases judged by either the reporting medical investigator or the sponsors as having a reasonable suspected causal relationship to an IMP (i.e. possibly, probably or definitely related to an IMP) will qualify as adverse reactions.

**Unexpected Adverse Reaction:** An adverse reaction, the nature or severity of which is not consistent with the applicable product information (*e.g.*, Investigator's Brochure for an unapproved investigational medicinal product) is considered as an unexpected adverse drug reaction.

**Serious Adverse Event (SAE):** An SAE is an AE that results in any of the following outcomes, whether or not considered related to the study intervention.

- Death (i.e., results in death from any cause at any time)
- Life-threatening event (i.e., the volunteer was, in the view of the investigator, at immediate risk of death from the event that occurred). This does not include an AE that, if it occurred in a more serious form, might have caused death.
- Persistent or significant disability or incapacity (i.e. substantial disruption of one’s ability to carry out normal life functions).
- Hospitalisation, regardless of length of stay, even if it is a precautionary measure for continued observation. Hospitalisation (including inpatient or outpatient hospitalization for an elective procedure) for a pre-existing condition that has not worsened unexpectedly does not constitute a serious AE.
- An important medical event (that may not cause death, be life threatening, or require hospitalization) that may, based upon appropriate medical judgment, jeopardize the volunteer and/or require medical or surgical intervention to prevent one of the outcomes listed above. Examples of such medical events include allergic reaction requiring intensive treatment in an emergency room or clinic, blood dyscrasias, or convulsions that do not result in inpatient hospitalization.
- Congenital anomaly or birth defect.

**Serious Adverse Reaction (SAR):** An adverse event (expected or unexpected) that is both serious and, in the opinion of the reporting investigator or sponsors, believed to be possibly, probably or definitely due to an IMP or any other study treatments, based on the information provided.

**Suspected Unexpected Serious Adverse Reactions (SUSARs):** A SUSAR is a SAE that is unexpected and thought to be possibly, probably or definitely related to an IMP.

## Causality assessment

For each AE, an assessment of the relationship of the AE to the study intervention(s) was undertaken. The relationship of the adverse event with the study procedures was categorized as unrelated, unlikely to be related, possibly related, probably related or definitely related. An intervention-related AE refers to an AE for which there is a possible, probable or definite relationship to the study intervention. The investigator used clinical judgment to determine the relationship. Alternative causes of the AE, such as the natural history of pre-existing medical conditions, concomitant therapy, other risk factors and the temporal relationship of the event to vaccination or CHMI was considered and investigated.

## Malaria Diagnosis

Diagnosis of malaria infection following CHMI was defined as positive thick film microscopy (at least one morphologically normal malaria trophozoite seen in 200 high-power (1000x) fields) by one or more experienced microscopists in a patient with symptoms suggestive of malaria.

Real time qPCR for *P. falciparum* was simultaneously performed, although Investigators (except the Chief Investigator) were blinded to the results. In the event of a positive thick film for malaria parasites in an asymptomatic volunteer, the investigators were un-blinded to the most recent PCR results for that subject only, and malaria treatment initiated only if any available PCR result for that subject had been measured as >500 parasites/ml. If all available PCR results for this subject were <500 parasites/ml, treatment was delayed until either they developed a further positive thick film in the presence of symptoms suggestive of malaria infection, or the volunteer has a further positive thick film with a PCR measurement above 500 parasites/ml.

In the event that a subject presented with symptoms or signs which were strongly suggestive of malaria infection despite having a negative blood film, investigators were un-blinded to the most recent qPCR results, and treatment initiated if any result exceeded 500 parasites/ml.

## *Ex-vivo* Interferon-γ (IFN-γ) Enzyme-Linked Immunosorbent Spot (ELISPOT) assays

*Ex vivo* (18 hour stimulation) ELISPOT assays were performed using Multiscreen IP ELISPOT plates (Millipore), human IFNγ SA-ALP antibody kits (Mabtech) and BCIP NBT-plus chromogenic substrate (Moss Inc). Cells were cultured in RPMI (Sigma) containing 1% sterile filtered Penicillin-Streptomycin (Sigma), 1% L-Glutamine (Sigma) and 10% heat-inactivated, sterile-filtered foetal calf serum, previously screened for low reactivity (Labtech International). Antigens were tested in triplicate with 250,000 PBMC added to each well of the ELISPOT plate. Plates were counted using an AID automated ELISPOT counter (AID Diagnostika GmbH, algorithm C), using identical settings for all plates and counts were adjusted only to remove artefacts. Responses to the negative control were always < 85 SFC per million PBMC. Responses were considered positive if four times greater than the negative control for the corresponding sample.

Figure 3C includes data from two previous clinical trials of ChAd63 MVA ME-TRAP [1, 2]. Assays were performed in the same laboratory using the same reagents and Standard Operating Procedures to ensure comparability between studies.

## Peptides for T cell Assays (Tables S6-S8)

TRAP peptides were 20 amino acids in length, overlapping by 10 amino acids (Neopeptide), assayed in 6 pools of 7-10 peptides at 10 μg/mL. CSP peptides were 15 amino acids in length, overlapping by 11 amino acids, assayed in 3 pools of 3-12 peptides at 10 μg/mL. Responses were averaged across triplicates, responses in unstimulated (negative control) wells were subtracted and then responses in individual pools were summed for each strain of the TRAP antigen or for CSP. ME responses were assayed in a single pool and peptide pool configurations are shown in Tables 14, 15, 16. Staphylococcal enterotoxin B (0.02 μg/mL) and phytohaemmagglutinin-L (10μg/mL) were used as a positive control.

## Flow cytometry with Intracellular Cytokine Staining (ICS)

Flow cytometry for vaccine-induced responses to TRAP and CSP was performed at C-1. Samples for flow cytometry were stimulated in parallel with the *ex-vivo* ELISPOT using fresh PBMC. After overnight stimulation, samples were stained and acquired the same day on the Jenner Institute LSR II flow cytometer. Responses to CSP and TRAP were assessed using a single pool of peptides for each antigen. Peptide sequences are described in Tables S6 to S8. Details of antibodies used for staining are given in Table S9. A hierarchical gating strategy was applied for analysis (Figure S4).

Responses were assessed by a 9-colour staining panel on freshly isolated PBMC, in parallel with ELISPOT assays. Aliquots of 2 × 10^6^ PBMC in 1 ml of medium containing anti-CD28 and anti-CD49d at 1 μg ml−1 (eBioscience) and CD107a-PeCy5 (1:500, eBioscience) were stimulated with no antigen, a pool of 56 peptides spanning the T9/96 strain of the TRAP antigen (20mers overlapping by 10 amino acids, at 2 μg ml−1), a pool of 56 peptides spanning the 3D7 strain of the TRAP antigen (20mers overlapping by 10 amino acids, at 2 μg ml−1), a pool of 31 peptides spanning the CS antigen (15mers overlapping by 11 amino acids, at 2 μg ml−1) or a positive control, Staphylococcal enterotoxin B (Sigma, 1 μg/ml) in 5 ml polystyrene FACS tubes for 18 hours at 37°C and 5% CO2. Brefeldin A and Monensin, both at 1 μg/ml, were added for the last 16 h. Cells were incubated with a dead cell discrimination dye (AQUA 1:200, Invitrogen) for 20 minutes at room temperature. PBMC were permeabilised, then stained intracellularly at room temperature for 30 minutes with CD4-APC (1:25, eBioscience) CD14- and CD19-Pacific Blue (both 1:50, eBioscience), CD3-Alexa Fluor 700 (1:50, eBioscience), CD8-APC-Alexa Fluor 780 (1:10, eBioscience) and IFN-γ-FITC (1:100, eBioscience), IL-2-PE (1:50., eBioscience) and TNFα-Pe-Cy7 (1:500, eBioscience), then washed and fixed in 1% paraformaldehyde. Further details of monoclonal antibodies are given in Table S9 Compensation was performed using single-stained One-Comp beads (eBioscience) for monoclonal antibodies and ARC beads for AQUA (Life Technologies).

Acquisition was performed on the day of staining on a BD LSRII with median of 675,000 live CD3+ cells acquired (IQR 506,250-765,000) per sample. Data was prepared and analysis performed using FlowJo v9.6.2 (Treestar Inc). Cells were gated on lymphocytes, singlets, live CD3+, CD8-CD4+ or CD4-CD8+ and then IFNγ, IL-2, TNFa and CD107a. Dead cells (AQUA+), monocytes (CD14+) and B cells (CD19+) were excluded from the analysis. All SEB stimulated PBMC gave a cytokine response >1%. Responses to peptide were determined after subtraction of the response in the unstimulated control for each sample, and considered positive if the count was >20 and frequency higher than the autologous unstimulated control and the lower limit of detection (LLD CD4^+^ = 0.002, LLD CD8^+^= 0.002).

## IgG ELISAs

Anti-CS IgG was measured on serum samples collected on D0, D28, D42, D49, C-1, 35 days after CHMI (C+35) and C+90. Antibody responses to TRAP were measured on D0, D21, D28, D35, 42, 49, 56, C-1, C+35 and C+90 by IgG ELISA. Both assays were performed at the Jenner Institute, UK. Antibody responses to CS were also measured by IgG ELISA performed at the WRAIR International Reference Centre for Malaria Serology.

## Total IgG Enzyme Linked Immunosorbent Assay (ELISA) to TRAP

Recombinant TRAP antigen was produced by transient transfection of HEK293E cells, using a method similar to that previously reported. [5, 6] A transgene comprising the human tissue plasminogen activator secretory signal peptide fused in frame with the 3D7-clone TRAP ectodomain (lacking the native signal peptide, transmembrane domain and a run of PNP repeats stretching from P356 to P370) was codon-optimised for mammalian expression (Life Technologies). The transgene cassette was cloned using the InFusion enzyme system (ClonTech) into the pENTR LPTOS plasmid backbone, [7] in which expression of the transgene is driven by an intron-containing CMV immediate early promoter, with additional in-frame C-terminal biotin acceptor peptide and Strep(II) tags. [8] Four days after polyethyleneimine mediated transfection of HEK293E cells, supernatant was harvested and affinity purified on a Streptactin affinity column (IBA Lifesciences). The resulting protein was >90% pure, as demonstrated by Coomassie Blue stained SDS-PAGE (data not shown).

Nunc-Immuno 96 well plates were coated with 0.5μg/mL of TRAP antigen in carbonate-bicarbonate coating buffer and left overnight at 4°C. Plates were washed 6x with PBS-Tween (PBS/T), then blocked with 1% BSA in PBS/T for 1 hour at room temperature (RT). Serum was diluted in PBS/T containing 0.2% BSA at concentrations of 1:100, 1:300, or 1:900, and added in triplicate. Serum samples from days 0, 28, 56, 63, C-1, C+7 and C+90 were analysed. Plates were incubated at RT for 2 hours then washed as before. A secondary antibody (goat anti-human whole IgG conjugated to alkaline phosphatase, Sigma) was added at a dilution of 1:1000 in PBS/T 0.2% BSA for 1 hour at RT. After a final wash, plates were developed by adding 4-nitrophenyl phosphate in diethanolamine buffer (Pierce).

A positive reference standard (made from pooled TRAP-positive serum) was used on each plate to give a standard curve. It was added in duplicate at an initial dilution of 1:100 (in PBS/T 0.2% BSA) and diluted 2-fold 10 times, starting with an arbitrary value of 20 antibody units. 4 blank wells (zero antibody units) were also designated. The optical density (OD) values were then fitted to a 4 parameter standard curve using SOFTmax PRO software^.^. [9] An internal control was included on every plate in triplicate made up from a 1:400 dilution (in PBS/T 0.2% BSA) of the positive standard. OD was read at 405nm using an ELx800 microplate reader. Test sera antibody units were calculated from their OD values using the parameters estimated from the standard curve.

## Total IgG Enzyme Linked Immunosorbent Assay (ELISA) to CS [10]

ELISA 96-well plates were coated with a synthetic peptide (Eurogentec) based on the repeat region of the PfCSP with the amino acid sequence CS(NANP)6C. The peptide was coated at a concentration of 0.2 μg/mL in a volume of 100 μL per well. Plates were placed inside a humidity chamber and incubated overnight (16 - 20 h) at 22°C. Plates were washed four times with 1xPBS (pH 7.4) containing 0.5% Tween-20 and blocked with 0.5% casein blocking buffer (Sigma) for 1 h at 22°C. Plates were washed four times and serially diluted samples were added and incubated at 22°C for 2 h. After washing four times, peroxidase labeled goat anti-human IgG (KPL) was added at a dilution of 1:4,000 and incubated at 22°C for 1 h. After washing four times, ABTS Peroxidase substrate (KPL) was added for development and incubated for 1 h at 22°C. The data were collected using Softmax Pro GXP, data were fit to a 4-parameter logistic curve, and the serum dilution at which the optical density was 1.0 (OD 1.0) calculated. To serve as a positive control, serum obtained from a volunteer participating in a Phase 1/2a challenge trial of R32NS181 formulated with alum was used. [11] The individual had anti-PfCSP antibodies but was not protected (*personal communication from WRAIR serology laboratory*). Samples were considered positive if the difference between the post-immunization OD 1.0 and the pre-immunization OD 1.0 (net OD 1.0) was > 50 and the ratio of post- immunization OD 1.0 to pre-immunization OD 1.0 (ratio) was > 2. For example, if the OD 1.0 was 150 post-immunization and 50 pre-immunization, the net OD 1.0 would be 100, and the ratio of OD 1.0 post-immunization to OD 1.0 pre-immunization would be 3. This would be considered positive. Kinetics of the NANP-specific response are shown for all volunteers receiving RTS,S/AS01B alone (groups 1 and 2 combined) and volunteers receiving RTS,S/AS01B with viral vectors (groups 3 and 4 combined) until the third vaccination where groups 2 and 4 received a fractional dose of RTS,S/AS01B and are shown separately (Figure 4A). In further analyses, groups 1 and 2 (RTS,S/AS01B only), and groups 3 and 4 (RTS,S/AS01B with viral vectors) were combined as there were no significant differences between these groups on these assays.

## CSP-specific IgG Avidity ELISA

IgG antibody avidity was assessed by sodium thiocyanate (NaSCN)-displacement ELISA. The assays were conducted as for total IgG ELISAs except that sera were individually diluted in casein to a level calculated to reach an OD405 of 1.0 (using total IgG EUs), and plated at 50uL/well in 16 wells of a 96 well plate. Plates were incubated for 2 hours at RT before chaotropic agent NaSCN was added in duplicate at increasing concentrations down the plate (0 to 7 Molar (M)). Plates were incubated for 15 mins at RT before washing, incubated with secondary antibody and developed with the same conditions as the total IgG assay. The concentration of NaSCN required to reduce the OD405 to 50% of that in wells where no NaSCN (=IC50) was added was used as a measure of avidity.

## Anti-MVA IgG ELISA

Anti-MVA ELISAs were conducted using serum taken at baseline (D0), D42 and C-1 for all volunteers. Pre-coated and pre-blocked 96-well ELISA plates were kindly donated by Dr. Huw Davies, University of California, Irvine. Plates were coated with MVA protein WR113/D8L, blocked with Casein/TBS, dried and stored at 4°C until use. Samples were diluted 1:200 in blocking buffer (1% Casein in TBS supplemented with 10% E. coli lysate). After incubating in blocking buffer at room temperature (RT) for 30 minutes to block anti-E. coli reactivity, samples were added to the plate in duplicate, 50uL/well. Plates were incubated for 45 minutes at RT before washing 6 times with PBS. Secondary antibody (goat anti-human IgG conjugated to Horseradish Peroxidase, ADI, H-HuG.211) was added 100uL/well at a dilution of 1:100 in PBS and plates were incubated at RT for 45 minutes. Plates were washed 6 times in PBS before adding 100uL of TMB substrate (ADI, 80091) per well and covering plates to protect from light. After 10 minutes, the reaction was stopped by adding 100uL/well of stop solution (ADI, 80101). Optical density (OD) was read at 450nm on an ELx800 microplate reader (Biotek) with Gen5 software (version 2.07).

## Parasite Quantitative PCR (qPCR)

*P. falciparum* qPCR was performed as previously described.[6] Blood was collected at baseline and at time points following CHMI for qPCR in 2.0ml tubes containing EDTA before being filtered to reduce white cell content. DNA was extracted from 0.5mL filtered blood using Qiagen Blood Mini Kit. 5μL of each extraction was used per assay (total eluate volume = 50μL) and was run in triplicate for qPCR (equivalent to 150μL blood directly assessed). Parasites per mL (p/mL) equivalent mean values were generated by a standard Taqman absolute quantitation, against a defined plasmid standard curve. This was conducted on an ABI StepOne Plus machine and v2.3 software using default Universal qPCR and QC settings, apart from the use of 45 cycles and 25μL reaction volume.

## Statistical Analysis

A statistical analysis plan (SAP) was drafted by the study statistician and can be found in the supplementary appendix to this article. Data was analysed using a variety of software including Microsoft Excel for Windows version 14 (Microsoft Corporation, Washington, USA), SPSS for Mac version 23.0 (IBM Corp., USA) and GraphPad Prism for Windows or for Mac version 6 (GraphPad Software Inc., California, USA). All tests were 2-tailed.

# References

1. Ewer KJ, O'Hara GA, Duncan CJ, et al. Protective CD8+ T-cell immunity to human malaria induced by chimpanzee adenovirus-MVA immunisation. Nat Commun **2013**; 4:2836.

2. Rampling T, Ewer KJ, Bowyer G, et al. Safety and High Level Efficacy of the Combination Malaria Vaccine Regimen of RTS,S/AS01B with ChAd-MVA Vectored Vaccines Expressing ME-TRAP. J Infect Dis **2016**.

3. Conroy RM, Pyorala K, Fitzgerald AP, et al. Estimation of ten-year risk of fatal cardiovascular disease in Europe: the SCORE project. Eur Heart J **2003**; 24:987-981003.

4. O'Hara GA, Duncan CJ, Ewer KJ, et al. Clinical assessment of a recombinant simian adenovirus ChAd63: a potent new vaccine vector. The Journal of infectious diseases **2012**; 205:772-81.

5. Crosnier C, Bustamante LY, Bartholdson SJ, et al. Basigin is a receptor essential for erythrocyte invasion by Plasmodium falciparum. Nature **2011**; 480:534-7.

6. Hodgson SH, Ewer KJ, Bliss CM, et al. Evaluation of the Efficacy of ChAd63-MVA Vectored Vaccines Expressing Circumsporozoite Protein and ME-TRAP Against Controlled Human Malaria Infection in Malaria-Naive Individuals. J Infect Dis **2015**; 211:1076-86.

7. Douglas AD, Williams AR, Illingworth JJ, et al. The blood-stage malaria antigen PfRH5 is susceptible to vaccine-inducible cross-strain neutralizing antibody. Nat Commun **2011**; 2:601.

8. Voss S, Skerra A. Mutagenesis of a flexible loop in streptavidin leads to higher affinity for the Strep-tag II peptide and improved performance in recombinant protein purification. Protein Eng **1997**; 10:975-82.

9. Miura K, Orcutt AC, Muratova OV, Miller LH, Saul A, Long CA. Development and characterization of a standardized ELISA including a reference serum on each plate to detect antibodies induced by experimental malaria vaccines. Vaccine **2008**; 26:193-200.

10. Epstein JE, Tewari K, Lyke KE, et al. Live attenuated malaria vaccine designed to protect through hepatic CD8(+) T cell immunity. Science **2011**; 334:475-80.

11. Rickman LS, Gordon DM, Wistar R, Jr., et al. Use of adjuvant containing mycobacterial cell-wall skeleton, monophosphoryl lipid A, and squalane in malaria circumsporozoite protein vaccine. Lancet **1991**; 337:998-1001.
